# The impact of touchscreen digital exposure on children’s social development and communication: a systematic review

**DOI:** 10.3389/fpsyg.2025.1613625

**Published:** 2025-10-13

**Authors:** Anastasia Misirli, Olga Fotakopoulou, Maria Dardanou, Vassilis Komis

**Affiliations:** ^1^Department of Educational Studies & Early Childhood Education, University of Patras, Patras, Greece; ^2^College of Psychology, Birmingham City University, Birmingham, United Kingdom; ^3^Department of Teacher Education and Pedagogy, UiT, The Arctic University of Norway, Tromsø, Norway

**Keywords:** touchscreens, social development, communication skills, peer interaction, cooperation, collaboration, early childhood

## Abstract

Touchscreen technologies—ranging from smartphones and tablets to interactive whiteboards and tabletops—are increasingly embedded in the daily lives of young children, shaping how they communicate and interact socially. The present systematic review aims to understand aspects of touchscreen use that support, hinder or explain changes in how young children (ages 1–6) develop social functioning skills such as peer interaction, cooperation, collaboration and communication. Specifically, it explores: (1) the types of touchscreen devices used, (2) their influence on peer interaction, cooperation and collaboration, (3) their effects on communication skills and (4) the developmental, educational, and policy recommendations emerging from the literature. A comprehensive search across Web of Science, ERIC, and Scopus yielded 365 studies, of which 82 met inclusion criteria following PRISMA guidelines. Using a theoretically grounded definition of social functioning and communication, we conducted a content analysis of empirical studies across social sciences, psychology, art and humanities, and computer science. Findings reveal a pervasive presence of touchscreen media in early childhood, with varied impacts shaped by device type, content quality, adult mediation, and contextual factors. This review offers evidence-based insights for educators, parents, and policymakers, emphasizing the value of interactive engagement, teacher training, public education efforts, and community-based approaches in promoting meaningful digital experiences. It stressed the importance of intentional, guided and contextual use of touchscreen technologies in early childhood and family settings. This review offers evidence-based insights for educators, parents, and policymakers, emphasizing the value of interactive engagement, teacher training, public education efforts, and community-based approaches in promoting meaningful digital experiences. It stressed the importance of intentional, guided and contextual use of touchscreen technologies in early childhood and family settings.

**Systematic review registration:**

Open Science Framework https://osf.io/9athz/?view_only=1fa731457d7e48248b7482bca02793f9.

## 1 Introduction

During this era of digital development, children are growing up surrounded by screens and digital devices. From smartphones and tablets to computers and televisions, digital exposure has become an integral part of their daily lives. While technology offers a wealth of educational and entertainment opportunities, it also prompts significant questions regarding its effects on children’s social development and communication skills (Massaroni et al., 2023; Ren, 2023; Rocha and Nunes, 2020).

Recent studies indicate that children under the age of 8 spend an average of 2.5 h per day on screen-based activities, with touchscreen devices accounting for a growing proportion of this time (Common Sense Media, 2025). This early and frequent exposure has raised concerns among researchers and health organizations about its potential impact on developmental outcomes. For instance, excessive touchscreen use has been associated with delays in expressive language development, reduced attention spans, and diminished quality of parent–child interactions (Madigan et al., 2019; Radesky et al., 2020). The World Health Organization (WHO) recommends that parents closely monitor screen time for older children, limiting it to no more than 2 h per day, while advising that infants under 1 year of age should not have any screen time (World Health Organization (WHO), 2019). The Australian Movement Guidelines for Children has addressed similar recommendations (Joshi and Hinkley, 2021). Screens are defined as the display interfaces of screen-based devices, which include mobile phones, tablets, televisions, and computers. Additionally, the American Academy of Pediatrics discourages media use by children younger than two and recommends limiting older children’s screen time to no more than 1 or 2 h a day (American Academy of Pediatrics, 2022).

The early years of life, particularly from birth to age six, are a critical period for the development of social and communication skills. During this time, children form foundational abilities in interacting with others, expressing themselves, and understanding social cues—skills that are essential for later academic and personal success (Institute of Medicine and National Research Council, 2015). Given the increasing presence of touchscreen technologies in young children’s environments, it is vital to understand how these tools influence early developmental trajectories. Therefore, this review focuses specifically on children aged 1–6 years, aiming to investigate the impact of touchscreen use on their social development and communication skills. By examining the relationship between touchscreen interaction and these key developmental domains, the study seeks to generate evidence-based insights and practical recommendations for parents, educators, and other stakeholders involved in early childhood care and education.

Screens are used for various activities such as social media interaction, gaming, and educational tasks (Smahel et al., 2020). Touchscreens refer to digital devices with a tactile-based interface that allows young children to interact with digital content through touch (Taherian Kalati and Kim, 2022). Research indicates that these devices can facilitate personalized, flexible, and mobile learning experiences, making them valuable tools in both formal and informal educational settings (Liu and Hwang, 2021; Lovato and Waxman, 2016; Russo-Johnson et al., 2017). The lightweight design and intuitive interface of touchscreens enable even very young children to engage meaningfully with educational apps and content (Kucirkova et al., 2019). The literature refers often to children’s “screen time” which is that time spent using an electronic device that has a screen, such as: a computer, television, game console, tablet, or cell phone (Hu et al., 2020) and often refers to passive time use from children. Children’s well-being has been associated with the use of screens (OECD, 2018; Twenge, 2019). Bustamante et al. (2023) suggested that screen time should be included as an active time during which parents or caregivers and gaming must be present. UNICEF (2017, p. 100) underlined that “more attention should be given to the content and activities of children’s digital experiences – what they are doing online and why – rather than strictly to how much time they spend in front of screens.” However, much of the research on screen time to date has focused on TV watching (Beatty and Egan, 2020).

Moreover, according to a study by Caballero-Julia et al. (2024), the healthy use of screens by children is significantly influenced by parents and teachers possessing the appropriate tools to mitigate the risks associated with excessive screen exposure by fostering digital literacy from an early age and enhancing digital awareness and education among parents. In a similar vein, Liu and Hwang (2021) reported that 35% of research focused on the use of touchscreen mobile devices to enhance young children’s language skills and that cognitive development in children has received the greatest emphasis among various research topics as young children generally enjoy using touchscreen mobile devices for learning. Additionally, Mâţa and Clipa (2020) examined the concerns of teachers and parents regarding young children’s use of these devices, alongside the discussions of future trends in integrating technology into early childhood education.

Research highlights that early exposure to multimodal technologies can shape children’s social development and communication skills, presenting both opportunities and challenges (Chaudron et al., 2018). One of the most critical aspects of social development in early childhood is the acquisition of language skills. A study contacted by Liu et al. (2024) suggests that technology, particularly when used in interactive and engaging ways, can foster language development. Digital platforms that encourage storytelling, role play, and collaborative activities can enhance communication competence among young children (Rahiem, 2021; Undheim, 2021). However, concerns arise regarding passive consumption of content, which may hinder language development if not balanced with active, meaningful interactions (Clemente-Suárez et al., 2024).

Moreover, digital exposure influences cultural awareness and identity formation in young children. As children engage with diverse narratives and perspectives through digital media, they develop a broader understanding of the world around them. This exposure can promote empathy and multicultural awareness, important components of social development (OECD, 2023). However, it’s vital to recognize that not all digital content is created equal; the quality, context, and pedagogical approach to technology use can significantly impact outcomes (Livingstone et al., 2024). The Norwegian government established the Screen Use Committee to enhance the evidence on how children’s and adolescents’ screen usage during kindergarten, school, and leisure activities impacts their health, quality of life, learning, and development and has formulated recommendations aimed at promoting balanced, safe, and healthy screen usage (Norwegian Ministry of Education, 2024). Nevertheless, the Committee has not concluded that there is a need to take strong measures at the national level regarding the banning of screens in schools or kindergartens in Norway (Norwegian Ministry of Education, 2024).

Blum-Ross and Livingstone (2016) and Chaudron et al. (2018) examined how screen interactions can influence children’s social skills, particularly when these interactions are centered around shared screen experiences. Arnott (2016) and Fotakopoulou et al. (2020) explored how screen time is both shaped by and shapes the various environments in which a child develops, with implications for communication and social development. Additionally, Xie et al. (2018) study touchscreen use and children’s learning outcomes, finding mixed results—some studies indicated that touchscreens could support cognitive development, while others found no significant evidence of such benefits.

During early childhood, children’s social lives develop along two closely connected paths. One is the path of socialization, the process by which children acquire the standards, values, and knowledge of their society. The second path is personality development, in which children develop their unique patterns of feeling, thinking, and behaving in a variety of contexts and circumstances (Lighfoot et al., 2018). In traveling these paths, children along with significant others in their lives play an active role in co-constructing the course of development (Hutto, 2008). They interpret and select from the various socializing messages they receive, becoming conversant with their culture’s funds of knowledge and rules of behavior. Social development refers to the process by which children learn to interact with others and develop relationships, behaviors, and social skills (Siegler et al., 2024). Social skills refer to the abilities we use every day to interact and communicate with others. They include verbal and non-verbal communication, such as speech, gestures, facial expressions, and body language (Bailey et al., 2019). Communication is defined as learning to express thoughts and emotions effectively, both verbally and non-verbally (Jones et al., 2019).

A child has strong social skills if they understand how to behave in social situations and grasp both written and implied rules when communicating with others. Securely attached children tend to develop better social skills than their peers who are not securely attached (Dwyer et al., 2010). This link likely arises from both the early and continuing effects of parent–child attachment on the quality of social behavior, as well as children’s working models of relationships (Shomaker and Furman, 2009). However, it is also possible that individual characteristics of the child, such as sociability, influence both the quality of attachments and their relationships with peers. According to a study conducted by Herodotou (2017), the interactive and social features of touchscreen media introduce new forms of prosocial behavior that warrant exploration and includes investigating how specific characteristics of the media or certain applications may influence children’s emotional competencies, as well as how children engage with application characters, potentially demonstrating empathy or exhibiting acceptance or rejection of others.

Understanding the importance of sharing, taking turns, and cooperating with others in a social setting is crucial throughout childhood. Positive behaviors such as cooperation and sharing are essential for human society, and children engage in these behaviors from an early age (Hermes et al., 2016). From the end of the first year of life, children interact with peers around toys regularly (Endedijk et al., 2020). In their peer interactions, they display affiliative behaviors such as offering toys to each other, as well as antagonistic behaviors such as claiming or taking away toys. By the end of the second year, toddlers begin to cooperate with each other, as their play activities often unfold around a common goal (Brownell, 2011). In the early years, children encounter the community outside their family first at kindergarten, and the social skills come into prominence with this encounter. In this new environment, children have to acquire some new social skills that are crucial for their future development and adjustment to the world. The early years are usually perceived as an introduction for a child to the world of peers and peer relationships.

In our study, we approach communication as a concept that includes not only verbal talk and gesture but also other bodily actions (embodied communication) that are directed at the touchscreen or other artifacts. Dourish (2001) introduced the concept of embodied interaction, which refers to the creation, manipulation, and sharing of meaning through engaged interaction with artifacts. We see embodied interaction with touchscreens as communication as it also involves how touchscreens have been used as tools to stimulate thought and action. Touchscreen technologies have become increasingly prevalent in children’s lives, providing new avenues for social interaction and communication. These devices allow children to engage with digital content in ways that can enhance their social skills and cognitive development (Elkind, 2015). The use of touchscreens can support learning through interactive apps that promote cooperation, sharing, and other social behaviors (Neumann and Neumann, 2015). This way of experiencing the world through the body is at the heart of the theory of embodied cognition, the notion that our knowledge and representations of concepts are a direct result of our physical experience with the environment (Wellsby and Pexman, 2014). Embodied cognition has changed the way we see the human mind and how we understand children’s learning of language, communication, and concepts (Reggin and Pexman, 2021).

This systematic review, conducted in accordance with PRISMA (Preferred Reporting Items for Systematic Reviews and Meta-Analyses) guidelines, investigates the impact of touchscreens on the social development and communication skills of young children. The review specifically targets the age group of 1–6 years, a critical developmental window during which foundational social and communicative abilities are rapidly emerging. By synthesizing current evidence, the study aims to determine whether touchscreen device use during these formative years is associated with positive or adverse outcomes, particularly in real-world social interactions and expressive and receptive communication. The review provides a structured and comprehensive analysis of the literature to clarify the extent, nature, and consistency of these associations.

The research questions that guided the systematic review were as follows:

- RQ1: What types of touchscreen devices are most used by children 1–6 years old in the studies under review, and how do these differ in design and functionality?
- RQ2: How does the use of touchscreens influence young children’s social development (including their ability to engage in peer interactions, cooperation, and collaboration)?
- RQ3: What are the effects of touchscreen usage on young children’s communication skills?
- RQ4: What recommendations have been identified in the literature regarding touchscreen use among young children, from developmental, educational, and policy perspectives?

## 2 Methods

### 2.1 Study design

A statistical analysis was conducted and identified the specific characteristics of the reviewed studies published over the last decade globally, including the predominant types of touchscreen devices examined. Additionally, a content analysis of the findings was carried out to explore the influence of touchscreens on young children’s social development, its effects on their communication skills, and to synthesize recommendations from three different perspectives (developmental, educational, and policy).

### 2.2 Participants

The majority of studies reviewed focused on typically developed children (*n* = 29) or parents and their children (*n* = 17) and examined their use of or practices with touchscreens devices. A smaller number of studies focused solely on parents (*n* = 13), including one involving grandparent, and their practices exploring their touchscreen related-practices time spent, or shared activities with children. Initial evidence also indicates that touchscreen use, and related contextual activities were mostly supported by children and educators (*n* = 11), with a few studies involving only educators (*n* = 3) and a single study (*n* = 1) involving by children, educators and parents collaboratively.

### 2.3 Interventions

The majority of research designs employed surveys and observational techniques, including video recordings or fieldnotes (*n* = 40). Additionally, 15 studies applied interviews either semi-structured or conducted in focus groups. Only a few studies (*n* = 5) implemented experimental designs typically involving the use of apps or structured play sessions. The remaining studies adopted a mixed-methods approach.

### 2.4 Systematic review protocol

To ensure a thorough literature examination, we implemented the systematic review procedure proposed in the PRISMA statement (Page et al., 2021). According to the guidelines of the PRISMA method, we reported on the relative flow diagram the phases of a review process, including the identification, screening, eligibility assessment, and eligible selected articles. To enhance the scope and relevance of our analysis and achieve significant impact, we initially included studies from diverse subject areas such as social sciences, psychology, art and humanities, and computer science, published in English, French, Greek, and Nordic languages. However, after preliminary trials and an additional independent search from each one of the first three authors, the results proved insufficient to meet the inclusion criteria since the results yielded no empirical studies. Consequently, we refined our criteria and narrowed down the final search to records of empirical studies published in English to ensure consistency and quality of our findings. Three authors worked on the screening and coding process. They conducted weekly meetings to discuss the process of selection.

### 2.5 Search strategy

We conducted a comprehensive literature search covering the period from January 2014 to December 2024 to capture all relevant original empirical studies published in peer-reviewed scientific journals over the past decade. The search strategy was systematic and guided by a set of detailed, pre-defined search terms, along with clearly established inclusion and exclusion criteria. These criteria were developed using the PICO framework (Population, Intervention, Comparison, and Outcome) to ensure methodological rigor and relevance. Additionally, the review protocol was pre-registered on the Open Science Framework to enhance transparency and reproducibility.^1^ As shown in Table 1, we present all the terms used in our search strategy. Searches were performed across three electronic online databases, including Web of Science (WoS), ERIC, and Scopus, to capture a wide array of peer-reviewed articles. Another reason we decided on these digital databases is because of their coverage across the different disciplines of interest. Initially we sought to establish precise search parameters, including key terms and phrases related to our research topic, such as “Digital media” AND “Age” AND “Education level” AND “Social Development” AND “Communication skills.” Yet, no other systematic review has been published to be used as a reference point. We tried all possible extensions of keywords to narrow down the results. We utilized Boolean operators to enhance our search, combining terms with “AND” and “OR” and “AND NOT” ensuring a focus on social development—particularly peer interaction and cooperation—while also capturing all types of communication skills and its features with our scope. The initial search string with terms in each topic included was SS = (“touchscreen*”) AND (“child*”) AND NOT (“youth” OR “adolescent*” OR “elderly”) AND (“school” OR “preschool*” OR “kindergarten*” OR “early years”) AND NOT (“primary school” OR “middle school” OR “elementary school” OR “secondary school” OR “university” OR “college” OR “higher education”) AND (“social” OR “cooperation” OR “collaboration” OR “peer” OR “interaction”) AND (“communication”) AND NOT (“disability” OR “disorder” OR “implant” OR “autism” OR “spectrum” OR “dystonia” OR “health”). After the first cycle of searching that did not identify sufficient registers in either of the three databases, we came to the conclusion that there was a tendency in the literature to use terms for the type of device (smartphone, tablet) or even the term used by a certain brand (iPad) rather than their broader touchscreen category. Thus, we reformulated the query by replacing the term “touchscreen” with more specific terms for these devices taken from their marketized names. Moreover, as suggested by Webster and Watson (2002), we conducted a *backward search* to identify relevant work cited in similar reviews but without much success. Only reports were mainly obtained by our search, which focused on screen use and children’s health (mental & physical), safety, and wellbeing (House of Commons Education Committee, 2024; POSTnote365, 2020). In Appendix A, there is a presentation of search strings and queries of the three databases.

**Table 1:** Search strategy.

PICO	Search string
Population	(“child*” AND “school” OR “preschool*” OR “kindergarten*” OR “early years”) AND
Intervention	(“touchscreen*” OR “smartphone*” OR “tablets*” OR “i-Pad*”) AND
Outcomes	(“social” OR “cooperation” OR “collaboration” OR “peer” OR “interaction” OR “communication” OR “verbal talk” OR “eye-gaze” OR “gesture” ΟR “body language”)

### 2.6 Data sources

The research team initiated the collection of relevant studies across three electronic online databases: WoS, ERIC and Scopus. A specific set of inclusion and exclusion criteria was established to guide the present research, focusing on the publication year, publication type, subject areas, participants’ age, and education level (Table 2). The publication period was restricted to 2014–2024 primarily due to the limited number of earlier studies and the impact of two significant US reports published in 2022 and 2025—American Academy of Pediatrics (2022) and Common Sense Media (2025)—which might have influenced subsequent research on the topic. Additionally, article types such as reports, white papers, systematic reviews, proceedings, books and dissertations were excluded due to this. Papers focusing solely on academic learning and without empirical research were excluded. However, we decided to include a small number of theoretically significant works, which is a minor justified adaptation due to their conceptual nature and retained due to their relevance to review questions 2, 3 and 4. These articles did not report participant data, and this is reflected in the summary table (Appendix B) where ‘Not applicable’ is used in the sample size column.

**Table 2:** Inclusion and exclusion criteria.

Inclusion criteria	Exclusion criteria
IC1 Include only original empirical studies published in journals (articles full or short) and theoretically significant works	EC1 Exclude all scientific books and systematic reviews
IC2 Include only studies focused on social skills and communication development	EC2 Exclude all records sorted as conference proceedings, dissertations and thesis
IC3 Include only studies targeted in ages 1–6	EC3 Exclude all papers categorized reports, policy documents and white papers
IC4 Include only research written in English	EC4 Exclude all papers published in any other foreign language
IC5 Include only studies published after 1st January 2014	EC5 Exclude all papers published before 1 January 2014
IC6 Include only research items available to download	EC6 Exclude research conducted on special needs or other age groups

Articles eligible for inclusion were empirical studies published in peer-reviewed scholarly journals that would ensure validity and reliability. To ensure relevance, participants’ age was restricted to the keyword ‘child’ and combined with education level to match and cover precisely school or other settings. This choice was supported by search results that yielded many unrelated studies on ‘youth’, ‘adolescent’, and ‘elderly’ while trialing the keywords. The selected studies had to examine touchscreen digital devices and report findings on social development and/or communication skills. Hence, the subject areas we were to choose from in the databases WoS, ERIC and Scopus were those of social sciences, psychology, art and humanities, and computer science. Moreover, studies on special education (e.g., autism and dystonia), and health-related topics (e.g., depression, anxiety, implants) were excluded. Finally, only articles published in the English language were considered.

### 2.7 Data extraction

Three different investigators of the research group were assigned to each one of the three databases (WoS, ERIC, and Scopus) and followed the agreed upon search protocol and strategy. Each investigator performed a screening and reviewed the abstracts independently for the database that was assigned to. Data was extracted systematically to maintain consistency and accuracy across all included studies. At the beginning of the search, for about 2 weeks, we conducted trials for each electronic database to familiarize ourselves with the digital environment and its behavior; thus, we developed and confirmed the search strings. A shared spreadsheet file was used as the extraction form to record our data. Each member was assigned a specific equal space in this database, for which they were responsible. For each database, the following information was extracted and registered in gathering all key details, including study characteristics (article, author, year, type of publication, methodology and findings), population data (age range, education level), touchscreen device (tablet, iPad, smartphone) and the independent variables of social development and communication skills with all associated terms agreed (peer interaction, cooperation, collaboration and communication). To ensure the reliability and consistency of the data analysis, we employed a two-phase process and a combination of working interchangeably for the three databases. In the first phase, each author independently carried out the initial coding of their database. The second phase incorporated a second round of coding on the same dataset by the first author for the second and third authors’ datasets. The first author’s dataset was coded in the second round by the second author. To evaluate inter-rater consistency, Cohen’s Kappa (*κ*) statistic was calculated, yielding a value of approximately 0.83, indicating substantial agreement according to the interpretive scale proposed by Landis and Koch (1977). To validate and handle all extracted data accurately, we held weekly meetings to discuss and resolve discrepancies and reach a consensus. This approach ensured that the final codes represented a shared interpretation of the coding scheme. The rigor applied in this process supports the overall trustworthiness of the qualitative analysis and is consistent with establishing best practices in content analysis (Braun and Clarke, 2006). At the end of the process, we organized the different datasets into one final database and consulted the senior researcher (VK) for cross-verification before further analysis.

### 2.8 Data analysis

The first phase of the analysis involved a descriptive statistical analysis of the characteristics of the relevant studies during our understudy period. The next phase of analysis involved content analysis. Using content analysis, the content is coded for specific words, concepts or themes, allowing the analyst to draw inferences from emerging patterns (Krippendorff, 2004). Thus, we analyzed the qualitative data collected from the articles by creating categories of analysis based on the themes of our research questions. Data coding was carried out in phases through an iterative process to identify meaningful patterns. The first author performed coding and classification with the supervision of the other authors. Initially, all authors grouped codes that conveyed similar ideas, enabling us to recognize emerging patterns of social development and types of communication. These patterns were then categorized into broader categories and their frequency. This approach ensures that the identified categories are meaningfully aligned with our research questions and could effectively support further data analysis.

### 2.9 Study selection

**Figure 1:**
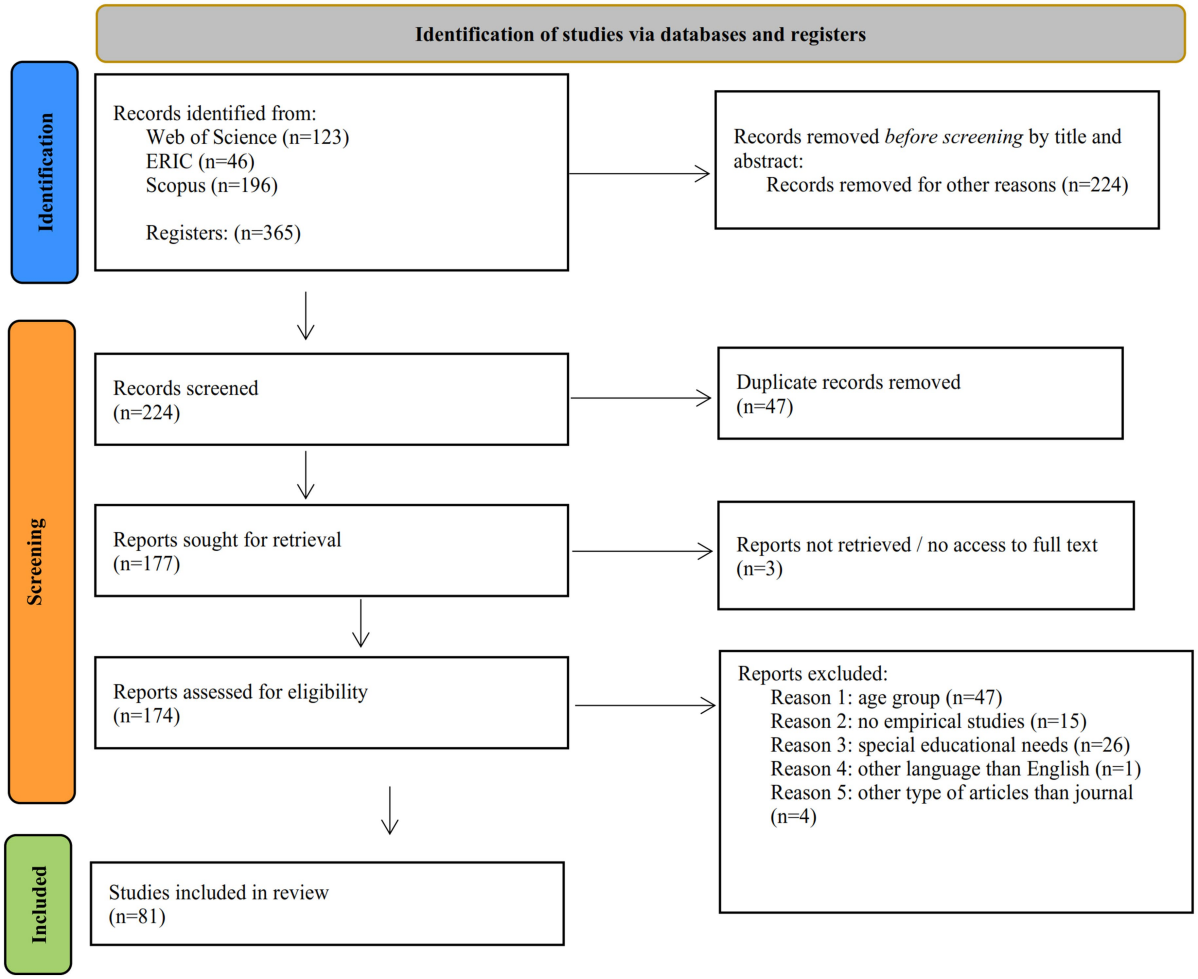
PRISMA flow diagram from Page et al. (2021).

In our review, the three different databases provided a solid collection of highly accredited peer-reviewed scientific articles. The WoS yielded one hundred twenty-three (123) records, whereas in ERIC, we retrieved forty-six (46) records, and lastly, in Scopus, we returned one hundred ninety-six (196) articles. Thus, we present a thorough and reliable selection of the latest developments in how touchscreens have been utilized through a decade, either in educational or family settings, to develop social and communication skills. A title and abstract screening were conducted at the first stage of the article selection process, and the article types were used as filters during the initial selection stage. Non-qualified publications were still returned to the records. After removing duplicates—the first screening applied—appearing in the different keyword searches within the same database and across databases; the number of qualified articles was reduced to 177. In that stage, only three reports were not retrieved and removed due to their limited access as full text and left us with 174 eligible articles. In the third screening stage, inclusion and exclusion criteria were applied by securing a full-text examination of each paper to exclude publications with non-relevant age groups, articles of other types than journals or empirical studies, special needs involved, and languages other than English. The research team assessed the methodology at the final stage to examine the validity of the research results. After applying the inclusion and exclusion criteria, we ended up with eighty-one (81) relevant articles for full-text review and analysis. In Figure 1, the number of records throughout the different phases of the review process is presented.

### 2.10 Assessment of risk of bias

When conducting the systematic review, we followed the ethical principles by the British Psychological Society (BPS, 2021) which are described below: (i) Transparency and Integrity: we clearly outlined the methods, scope and limitations of the review in accordance with PRISMA (Preferred Reporting Items for Systematic Reviews and Meta-Analyses) guidelines, (ii) Respect for Original Work: we properly cited all sources, acknowledging authorship and respecting intellectual property, and (iii) Avoiding Bias: we strived for impartiality in selecting and evaluating studies, ensuring comprehensive and balanced inclusion criteria.

## 3 Results

### 3.1 Study characteristics and background information

The authors examined each of the eighty-one (81) papers selected in detail and highlighted their characteristics (see Appendix B) including articles, authors, year and country of publication, sample size, setting, study design, key findings and types of touchscreen devices employed in the understudies.

### 3.2 Year published and country of origin

**Figure 2:**
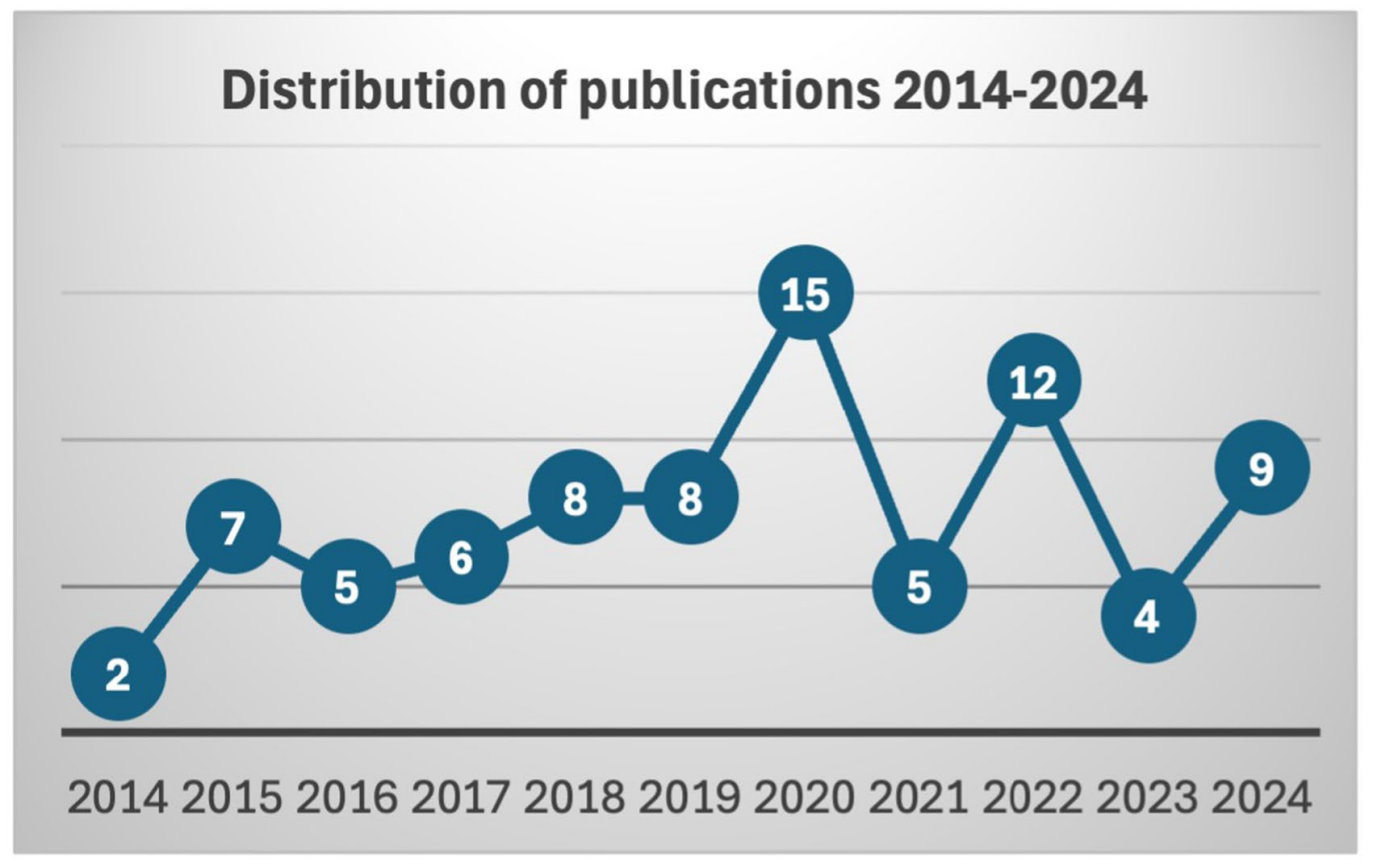
Distribution of publications during 2014–2024.

The review focused primarily on highlighting the distribution of the selected articles of the review through the decade covering from 2014 to 2024. As shown in Figure 2, the ‘journey’ began in 2014 with only two (02) publications. However, the following year shows an evolution of seven (07) publications. After that, the numbers again fell slightly, with four (05) in 2016, six (06) in 2017, and eight (08) in 2018, showing a quite dynamic technological trend. By 2019, the count reaches eight (08), and then there is a significant spike in 2020 with fifteen (15) publications, the highest across the aforementioned decade, followed by another substantial rise to twelve (12) in 2022. At that stage, we can recall that coincidentally 2020 is the year of the outbreak of the COVID-19 pandemic, which might have been the main factor influencing the academic community to publish against the increased exposure of children to touchscreens. Nonetheless, in 2021, there will be a drop back to five (05), as in 2016. The years 2020 and 2022 stand out as high points, while 2014 and 2023 had the least activity. Interestingly, 2023 falls to four (04), the lowest since 2016, before rising to nine (09) in 2024. The distribution suggests that more systematic and diverse research should be done.

**Figure 3:**
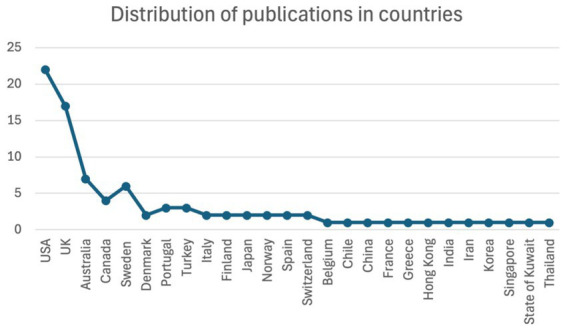
Distribution of publications in countries.

Another highlight of the review focused on showing the distribution of publications across countries, which presents a significant variation in research activity (Figure 3). The United States presented the highest number of outputs, with twenty-two (22) publications, followed by the UK with a slightly lower count and eighteen (17) outputs. Australia ranks third, contributing approximately seven (07) publications. A moderate output level is observed in Canada and Sweden, each with four (04) and five (05) publications, accordingly. In addition to these leading countries in scholarly output, there is a long list of countries, including Denmark, Portugal, Turkey, Italy, Finland, Japan, Norway, Spain and Switzerland, that have contributed between two (02) to three (03) publications. Even lower is the distribution in other parts globally, such as Belgium, Chile, China, France, Greece, Hong Kong, India, Iran, Korea, Singapore, the State of Kuwait, and Thailand, typically with one (01) publication each. While these figures highlight geographic trends in research activity, the specific factors underlying national contributions are not identified in this analysis. This disparity suggests an engagement with the topic across a diverse international group expanding beyond Europe. The current distribution highlights dominant research activity that emerged mainly from Western countries. On the other hand, there is a limited representation from Asian, Middle Eastern, and South American regions. The distribution suggests collaboration in future research to ensure more comprehensive and culturally diverse perspectives.

### 3.3 Distribution of touchscreen devices

**Figure 4:**
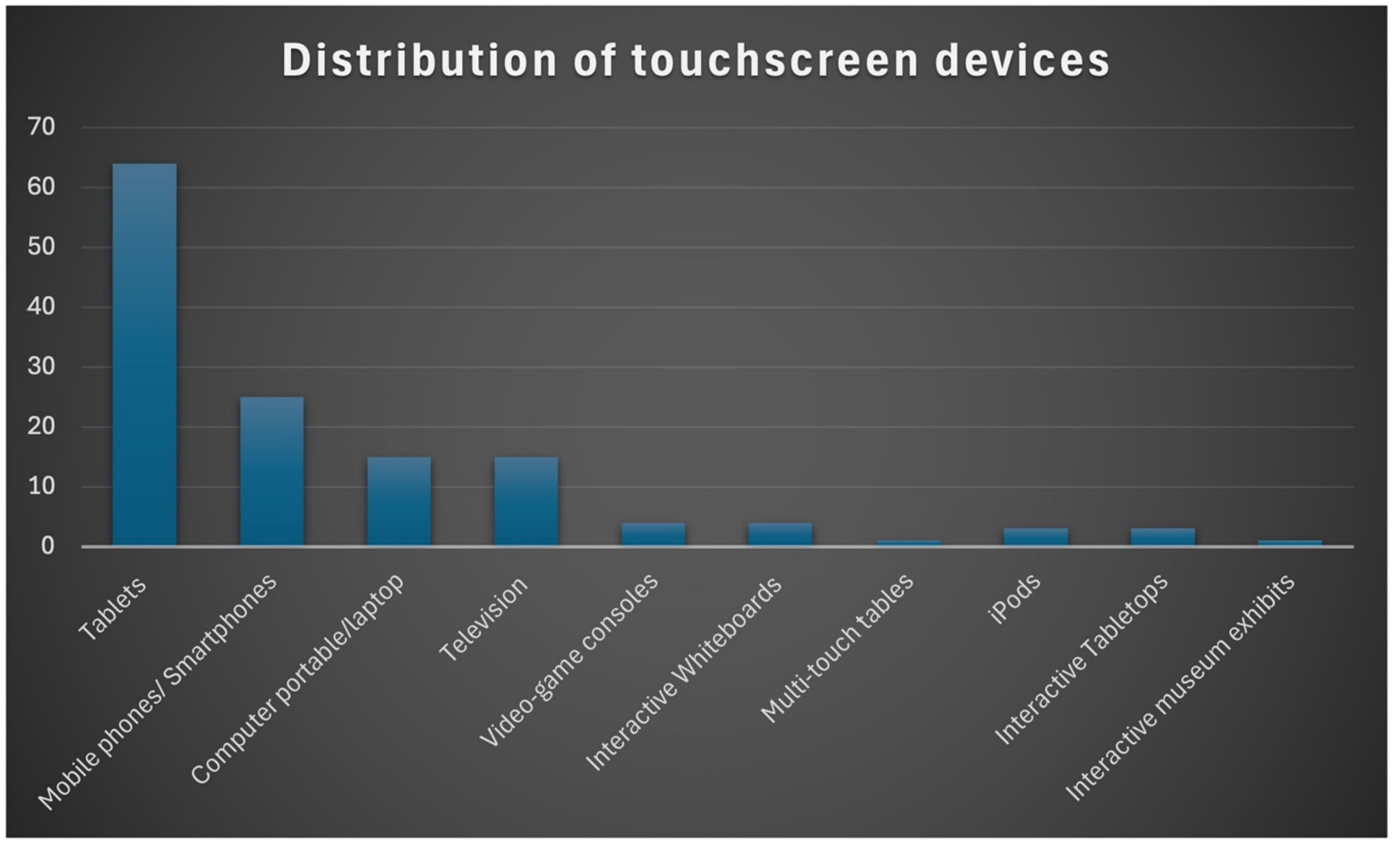
Distribution of touchscreen devices in the studies reviewed.

The classification of touchscreen devices in the selected reviewed studies reveals several notable trends (Figure 4). Interestingly, we make the distinction that studies using more than two devices may overlap and fall into multiple categories. Among these, ‘Tablets’ emerge as the most prominent, with sixty-four (64) studies using tablets (e.g., iPads or Android tablets). Similarly, the category of ‘Mobile phone/smartphone’, with twenty-five (25) papers. In contrast, the categories ‘Television’ and ‘Computer/laptop’ are each represented by fifteen (15) papers. The rest of the devices such as ‘Video-game consoles’, ‘Interactive Whiteboards’, ‘Multi-touch tables’, ‘iPods’, ‘Interactive Tabletops’ and ‘Interactive Museum exhibits’ fall into the category of a few studies from one (01) to four (04). Overall, the findings strongly focus on using tablets, particularly iPads, whether in school or family settings. A significant number of studies employed smartphones. A small number of studies reviewed proved to include more than one screen device but not exclusively touchscreen. Consequently, a more elaborate description of the prominent touchscreens’ design and functionalities is addressed in the first research question in the next section.

### 3.4 Synthesis of findings

In the following part, a content analysis of the 81 research studies was conducted. The findings are structured and presented according to the four research questions that guided our study.

RQ1. What types of touchscreen devices are most commonly used by children in the studies under review, and how do these differ in design and functionality?

The types of touchscreen devices more commonly used by children in the studies reviewed are tablets (e.g., iPads, Android tablets), smartphones (e.g., iPhones, Android phones), interactive whiteboards and interactive tabletops. The design of tablets allows for interactive engagement through touch, enabling children to use their fingers to navigate, select, and interact with the content. In terms of functionality, tablets support a wide range of applications that can cater to various educational and entertainment needs, promoting both individual and social interactions during play and learning activities (Samuelsson et al., 2021). Tablets are designed with larger screens, portability, and user-friendly interfaces, making them ideal for the participants of the studies. For instance, McCoy et al. (2017) utilized iPads for self-modeling with preschoolers, highlighting their effectiveness in engaging children during circle time in their classrooms. Similarly, Munzer et al. (2019) employed a 10-inch Samsung Galaxy tablet preloaded with electronic books. They conducted a videotaped, laboratory-based, counterbalanced study of 37 parent-toddler dyads reading across three book formats. The results indicated that both parents and toddlers verbalized less with electronic books and exhibited lower levels of collaboration when using electronic books compared to other formats.

Smartphones (e.g., iPhones, Android phones) were easily accessible to children, parents and educators, supported a variety of apps, including educational, creative and entertainment applications. Kushlev and Dunn (2018) conducted a study to investigate how smartphone use distracts parents from cultivating feelings of connection when spending time with their children. Although smartphones are designed to connect us with others, their use can create distractions that disconnect us from our immediate social environment. Frequent phone use led parents to feel more distracted, which in turn impaired feelings of social connection and the meaning that parents derived when spending time with their children. On the other hand, Demirsu (2020) found that smartphone mediated video-calls add new dimensions to communication, enhancing self-expression and bonding between migrant children with their grandparents. These calls facilitated visual performance, spatial sharing and spatial–temporal longing, while offering innovative ways to fulfill traditional grandparenting roles in a digitalized setting. Smartphones were selected for their high portability, accessibility, and ease of use for quick interaction and communication.

Interactive whiteboards provided the researchers in studies with large, fixed displays designed for classroom use, supporting teacher/researcher-led instruction, multimedia presentations and interactive sessions. Odgaard’s (2020) study significantly advances current research on technological tools in early childhood education. The activities examined were part of a broader, socio-culturally informed, design-based study conducted in collaboration with professionals and children in Denmark. The study explored how tablets and interactive whiteboards are used by children and professionals to co-produce and dialogically revisit multimodal books during the transition from day-care to school. By examining these activities over time, the study traced children’s sense-making of multimodal records across different contexts and periods. It highlighted how joint activities depend on participants’ initiation and maintenance of shared understanding, achieved through turn-taking procedures and sense-making utterances. The use of interactive whiteboards facilitated whole-class engagement with researchers, enabling both visual and interactive sessions as well as small group activities.

The large, horizontal surfaces of interactive tabletops supported multi-touch interactions and allowed multiple users to interact simultaneously, promoting collaborative activities and communication among participants. Interactive tabletops supported face-to-face interactions and equitable participation among users. Kubicki et al. (2015) developed an interactive tabletop application equipped with RFID technology. This tabletop, called TangiSense, is based on a multi-agent system that allows users to associate information with behaviors to manipulate tangible objects. The results from this study indicate that the tabletop attracts the interest of the children and teachers taking part in the experiment. This interest is characterized by more interactions and more error processing during the use of the tabletop specifically for the 4–6-year-old children. The youngest children (3–4 years old) from the sample of the study were more interested in manipulating objects.

These devices are chosen based on their specific strengths and the context in which they are used, whether for individual learning, group activities, or whole-class instruction. In the reviewed studies, the use of touchscreens devices supported the use of a wide range of educational apps (e.g., iWriteWords, Word Wagon HD, Park Math HD, Bugs and Numbers), creative apps (e.g., Doodle Buddy, Patterns Blocks, Toca Kitchen Monster, ABC Magnetic Alphabet), storytelling apps (e.g., Our Story, Skriv og Laes) and interactive features (e.g., touch gestures and responsiveness, multimedia capabilities). The use of touchscreen devices in the studies allowed touch interaction, portability and the ability to support individual and small group activities with the children.

RQ2. How does the use of touchscreens influence young children’s social development (including their ability to engage in peer interactions, cooperation, and collaboration)?

To address the second research question regarding the influence of touchscreen use on young children’s social development, including their ability to engage in peer interactions and cooperation, the research team systematically reviewed all relevant studies. Each study’s findings were recorded in relation to the second research question and validated by all team members. The data were then entered into NVivo 12 Plus, utilizing its auto-coding capabilities to identify recurring themes related to the impact of touchscreen use on young children’s social development. A content analysis was then performed (Neuendorf, 2017) and a coding scheme was developed to categorize the research studies’ findings. Three main categories emerged from the data: (i) positive influences, (ii) challenges and considerations, and (iii) mediating factors. Specifically, the reviewed studies highlighted several key advantages and disadvantages for children’s social development, along with some mediating factors. These findings are presented in Table 3 and analyzed below:

**Table 3:** Categories and subcategories of the effects of touchscreen usage on social development.

Categories	Positive influences	Negative influences	Mediating factors
Sub-categories	Collaborative learning	Reduced face-to-face interaction	Social scaffolding
	Peer interaction	Passivity in social interactions	Individual differences
	Social play	Parental disengagement	Cultural Perceptions
	Group activities	Communication skills	Educational settings
	Creative expression		

The first category of the Positive Influences of touchscreen uses on social development included five sub-categories:

*Collaborative Learning*: Multi-touch interfaces and tabletops facilitate collaborative learning by encouraging joint problem-solving and turn-taking. These devices support dialogues centered around learning activities, promoting social skills and cooperation (Burnett et al., 2020; Calhan and Göksu, 2024; Hatzigianni et al., 2018; Hegedus and Otálora, 2023; Kostyrka-Allchorne et al., 2017; Sakr, 2018).

*Peer Interaction*: Tablets and smartphones promote peer interaction and collaboration as children work together to complete tasks. This collaborative behavior enhances social engagement and helps children develop self-confidence and competence in digital play (Burnett et al., 2020; Karno and Hatcher, 2020; Studhalter et al., 2024).

*Social Play*: Video communication apps like Skype and Facetime on tablets facilitate social play, allowing children to interact with family members and friends, thereby supporting the development of social skills and maintaining social bonds (Karno and Hatcher, 2020; Verenikina et al., 2016).

*Group Activities*: Touchscreens, particularly iPads, foster increased peer collaboration in classroom settings. Children share knowledge, assist each other, and engage in group projects, enhancing their social interaction skills (Clarke and Abbott, 2016; McCoy et al., 2017; Odgaard, 2020; Sakr, 2019; Sinclair and Heyd-Metzuyanim, 2014).

*Creative Expression:* Digital play with touchscreens encourages children to share ideas and collaborate in creative activities, such as drawing and storytelling, promoting cooperation and social bonding (Booton et al., 2023; Disney and Geng, 2022; Little and Cheng, 2024; Peña et al., 2024; Sakr and Kucirkova, 2017; Shengjergji et al., 2024; Wohlwend, 2015).

The second category of Challenges and Considerations from the use of touchscreens on young children’s social development included four sub-categories which are listed below:

*Reduced Face-to-Face Interaction*: Excessive screen time can reduce opportunities for face-to-face peer interactions and cooperation, as children may become more engrossed in individual screen time rather than engaging with peers (Cruz et al., 2020; Shalani et al., 2023).

*Passivity in Social Interactions:* Unsupervised use of technology can lead to passivity in social interactions and difficulties in peer relationships, potentially resulting in isolation (Seo and Lee, 2017; Shawcroft et al., 2022).

*Parental Disengagement*: Single-user touchscreen displays can lead to parental disengagement, reducing opportunities for collaborative discussions and joint problem-solving, which are crucial for social development (Teichert, 2020).

*Communication Skills:* Touchscreen usage, particularly through electronic books, can negatively affect young children’s communication skills. Parents tend to focus on the technology rather than the story content, displacing meaningful dialogue and reducing educationally enriching experiences (Chaibal and Chaiyakul, 2022).

The third category of the content analysis, titled Mediating Factors included four sub-categories:

*Social Scaffolding:* Social demonstrations on touchscreen devices significantly improve children’s ability to replicate actions compared to ghost demonstrations. This enhances understanding and task completion, fostering skills like cooperation and joint attention (Antrilli and Wang, 2018; Hatzigianni and Kalaitzidis, 2018).

*Individual Differences*: Collaboration among children is influenced more by individual personality traits rather than the method of interaction (touchscreen or traditional). Some children may dominate the use of the device, leading to withdrawal from others, while others may use it as a tool to assist one another (Dempsey et al., 2018; Sinclair and Heyd-Metzuyanim, 2014).

*Cultural Perceptions*: Parents express concerns that excessive time spent on touchscreens may limit opportunities for outdoor play and face-to-face interactions, which are crucial for developing social skills (Dardanou et al., 2020).

*Educational Settings:* The presence and focus on iPads in early childhood settings can shape children’s interactions and behaviors, potentially reinforcing specific social behaviors and hierarchies (Lawrence, 2017).

Additionally, we analyzed with the use of NVivo the sentiments in the reviewed studies regarding the relationship between touchscreen usage and social development. We found 28 positive, 27 neutral, 21 mixed, and 17 negative references highlighting diverse perspectives on this topic (see Appendix C).

Overall, the studies suggest that touchscreens can both enhance and limit young children’s social development, depending on the nature of the interactions and the context in which they occur. When used thoughtfully and with proper guidance, touchscreens can support collaborative learning, peer interactions, and social skills development. However, excessive or unsupervised use may hinder face-to-face interactions and communication skills.

RQ3. What are the effects of touchscreen usage on young children’s communication skills?

To address the third research question regarding the influence of touchscreen use on young children’s communication skills, including their ability to engage in verbal and non-verbal interactions, the research team systematically reviewed all selected studies. Each study’s findings were recorded in relation to the third research question and validated by all team members. The data were then entered into NVivo 12 Plus, utilizing its auto-coding capabilities to identify recurring themes related to the impact of touchscreen use on young children’s communication skills. A content analysis was then performed (Neuendorf, 2017) and a coding scheme was developed to categorize the research studies’ findings. Two main categories emerged from the data: (i) positive influences and (ii) challenges. The reviewed studies highlighted several key advantages for children’s communication development and some considerable disadvantages, which are presented in Table 4 and analyzed below:

**Table 4:** Categories and subcategories of the effects of touchscreen use on communication skills.

Effects of use	Positive influences/challenges	Negative influences/challenges
Communication skills	Enhanced Parent–Child Conversations	Reduced Verbal Communication
	Collaborative Play	Delayed Language Development
	Support for Language Learning	Parental Distraction
	Multilingual Engagement	Less Face-to-Face Interaction
	Increased Confidence	Struggles with Social Cues

As it is demonstrated in Table 4, the content analysis revealed that touchscreen usage has been found to be linked to some Positive Influences that are organized into five subcategories and are listed below:

*Enhanced Parent–Child Conversations:* Some apps encourage verbal interactions and collaboration between children and parents or grandparents (Kucirkova and Sakr, 2015; Martinez, 2022; Moore and Adair, 2015; Zimmermann et al., 2017).

*Collaborative Play:* Touchscreens can facilitate cooperative activities that involve communication (Dardanou et al., 2020; Marsh et al., 2020; Yıldız, 2020).

*Support for Language Learning:* Interactive apps can help with vocabulary building and storytelling (Aarsand, 2019; Palmér, 2015; Troseth et al., 2016) or have negative effects (Gülay Ogelman et al., 2016; Taylor et al., 2022; Yoo and Smetana, 2022).

*Multilingual Engagement:* Some studies highlight how touchscreens can support bilingual or multilingual language development (Tavernier and Hu, 2020).

*Increased Confidence*: Children who are typically quiet may feel more comfortable engaging in discussions when using touchscreens (Disney and Geng, 2022; Yoo and Smetana, 2022).

The content analysis of the studies under review revealed that touchscreen usage is linked to some Challenges which are organized into five subcategories and are listed below:

*Reduced Verbal Communication:* Children may engage less in verbal interactions, relying more on gestures instead (Gross and Wang, 2024; Munzer et al., 2019).

*Delayed Language Development:* Excessive touchscreen time is linked to delays in expressive language and social communication (Booton et al., 2023; Hashmi et al., 2022).

*Parental Distraction:* Parents absorbed in their own devices and more frequently on their smartphones may reduce verbal engagement with their children (Morris et al., 2022; Shalani et al., 2023).

*Less/Limited Face-to-Face Interaction:* Children using touchscreens alone may have fewer opportunities for conversational practice (Moser et al., 2019).

*Struggles with Social Cues:* Some studies suggest that touchscreen use can make it harder for children to pick up social and emotional cues and decode social situations (Mallawaarachchi et al., 2024; Sinclair and Heyd-Metzuyanim, 2014).

Additionally, we analyzed with the use of NVivo the sentiments in the reviewed studies regarding the relationship between touchscreen usage and communication skills. We found 22 positive, 28 neutral, 22 mixed and 22 negative references, illustrating the varied perspectives on this topic (please see Appendix B).

Touchscreen usage can both support and hinder communication skills, depending on how it is used. Interactive, well-designed content and parental involvement seem to play a crucial role in determining its effects.

RQ4. What recommendations have been identified in the literature regarding touchscreen use among young children, from developmental, educational, and policy perspectives?

To address the fourth research question, the research team meticulously examined all studies to identify the recommendations proposed by the literature. Each study’s recommendations were recorded and subsequently imported into NVivo 12 plus for coding. We conducted content analysis and developed a coding scheme to categorize the content of the recommendations based on our research questions (developmental, educational, and policy perspectives). We analyzed the coded data to identify recurring themes, and in addition to the predefined categories, two more emerged from the data (practical and theoretical recommendations). The categories were further refined, resulting in four main categories, as described in Table 5.

**Table 5:** Categories and subcategories of recommendations for touchscreen use in young children.

Categories	Developmental perspectives	Educational perspectives	Policy perspectives	Practical recommendations
Sub-categories	Balanced screen time	High-Quality Educational Apps	Public Education Efforts	Monitor screen time
	Interactive engagement	Teacher facilitation	Guidelines for App Development	Community and environmental factors
	Guided use	Structured programs	Screen-Free Family Time	Educational content and parental involvement
	Parental involvement	Collaborative learning	Community-Based Approaches	Digital tools
	Empirical studies	Teacher training	Parent–Child Education	

The first category of recommendations is titled Developmental Perspectives and consists of five subcategories:

*Balanced Screen Time:* The researchers recommend limiting screen time and balancing it with traditional play and physical activities to support overall development (Okumura and Kobayashi, 2021; Pham and Lim, 2019).

*Interactive Engagement:* Encourage co-viewing and co-playing with adults to enhance meaningful learning outcomes (Formby, 2014; Jamil et al., 2017; Morris et al., 2022).

*Guided Use:* Provide appropriate support during touchscreen use to promote creativity and learning (Fleck et al., 2021; Sakr, 2019; Troseth et al., 2016).

*Parental Involvement:* Parents should regulate touchscreen use and screen time, set reasonable expectations, and engage in co-viewing or co-participation (Carson and Kuzik, 2021; Cruz et al., 2020; Kushlev and Dunn, 2018; Pasqualotto and Filosofi, 2023; Subiaul et al., 2015; Vartiainen et al., 2019).

*Empirical Studies:* Further empirical studies are needed to provide concrete guidelines for parents (Gülay Ogelman et al., 2016; Carson and Kuzik, 2021; Hatzigianni and Kalaitzidis, 2018; Kostyrka-Allchorne et al., 2017; Seo and Lee, 2017).

The second category of recommendations confirmed by the content analysis is Educational Perspectives and it consists of five subcategories:

*High-Quality Educational Apps:* The researchers of the reviewed studies recommend selecting apps that promote interaction and are educationally beneficial (Dashti and Habeeb, 2020; Kucirkova and Sakr, 2015).

*Teacher Facilitation:* Teachers should also actively facilitate touchscreen-based collaboration and integrate digital tools into pedagogical strategies (Eutsler and Trotter, 2020).

*Structured Programs:* They recommend the implementation of structured programs in schools that promote hands-on learning experiences alongside digital activities (Hatzigianni et al., 2018; Shalani et al., 2023; Yoo and Smetana, 2022).

*Collaborative Learning:* Researchers also advise designing tablet games that support collaborative learning by incorporating shared goals and meaningful interactions (Eutsler and Trotter, 2020; Munzer et al., 2019; Roldán-Álvarez et al., 2020).

*Teacher Training*: Teachers should receive training to effectively integrate technology into the classroom (Gülay Ogelman et al., 2016; Kubicki et al., 2015; Miller, 2018; Peña et al., 2024).

The third category that the content analysis confirmed is Policy Perspectives and consists of five subcategories:

*Public Education Efforts:* Researchers in the reviewed studies recommend improving public education efforts to inform parents about the benefits and risks of touchscreen devices in early childhood learning (Shtulman and Checa, 2017).

*Guidelines for App Development:* They also recommend the development of guidelines for app developers to ensure educational and developmental standards are met (Dore and Dynia, 2020; Taylor et al., 2022; Van den Bulck et al., 2016; Vartiainen et al., 2019; Zimmermann et al., 2017).

*Screen-Free Family Time:* They encourage screen-free family time to improve early childhood development (Carson and Kuzik, 2021; Teichert, 2020).

*Community-Based Approaches:* The researchers also advise the implementation of supervised after-school programs to encourage children to spend less time on screens and more time outdoors (Hegedus and Otálora, 2023; Nacher et al., 2020; Quinones and Adams, 2021).

*Parent–Child Education*: They also recommend educating parents on appropriate smart device usage and reducing absolute usage time to improve language development (Kushlev and Dunn, 2018).

The fourth category that emerged from the data is labeled Practical Recommendations and consists of four subcategories:

*Monitor Screen Time:* Parents should monitor screen time more effectively, establish clear usage guidelines, and engage in interactive activities to balance digital consumption (Chaibal and Chaiyakul, 2022; Hashmi et al., 2022; Moon et al., 2019; Shawcroft et al., 2022).

*Community and Environmental Factors:* Researchers in the reviewed studies also recommend community-based approaches and creating safe, engaging outdoor spaces can help reduce sedentary screen time (Moon et al., 2019; Nolan and Moore, 2024; Rodrigues et al., 2022).

*Educational Content and Parental Involvement:* They also recommend that educational content should be balanced with traditional play and interaction, and parents should be involved in media use (Calhan and Göksu, 2024; Dardanou et al., 2020; Marsh et al., 2020; Moser et al., 2015; Otterborn et al., 2019).

*Digital Screening Tools:* Lastly, some research studies propose considering digital screening tools as a feasible option for developmental assessments in pediatric clinics (Aarsand, 2019).

These themes highlight the importance of a balanced approach to touchscreen use, integrating technology thoughtfully into educational practices, and providing clear guidelines and support for parents and educators.

## 4 Discussion

The findings from the reviewed studies highlight the diverse range of touchscreen devices used by young children, including tablets, smartphones, interactive whiteboards, and interactive tabletops, each with unique design and functionality. Tablets, being the most used devices, offer interactive and portable learning opportunities that support both independent and collaborative activities (McCoy et al., 2017; Munzer et al., 2019). Research on children’s media use aligns with these findings, emphasizing the importance of co-use with parents or caregivers to enhance learning outcomes (Strouse et al., 2013). However, concerns have been raised regarding solitary use, as it may displace important psychosocial skill-building activities, potentially leading to behavioral difficulties (Mallawaarachchi et al., 2024; Munzer et al., 2019).

Smartphones, though widely accessible, present both opportunities and challenges for young children’s development. While they facilitate communication and bonding, such as through video calls with distant family members (Demirsu, 2020), they can also serve as distractions that interfere with meaningful parent–child interactions. Although smartphones are designed to connect us with others, such smartphone use may create a source of distraction that disconnects us from the people in our immediate social environment (Kushlev and Dunn, 2018). This supports existing literature on mobile touchscreen use, which suggests that parental phone use may impair social connection and engagement with children (Munzer et al., 2019). Furthermore, parents who rely on mobile devices as behavioral management tools often report unintended consequences, such as increased behavioral difficulties in children (Mallawaarachchi et al., 2024).

Interactive whiteboards and tabletops were used in fewer studies but were shown to enhance collaboration and engagement, particularly in group learning environments (Kubicki et al., 2015; Odgaard, 2020). These findings are consistent with research on interactive technologies in education, which emphasize their role in fostering social learning and cooperative skills (Hatzigianni et al., 2018; Hegedus and Otálora, 2023). The literature suggests that shareable, networked, and interactive devices like these may be more conducive to collaboration compared to handheld devices, which are more frequently used in solitary contexts (Mallawaarachchi et al., 2024). During the last 10 years, several researchers have utilized interactive tabletops as a learning medium. Different questions have been studied, and the main one has been whether the interactions supported by interactive tabletops may enhance user collaboration (Burnett et al., 2020; Calhan and Göksu, 2024; Kostyrka-Allchorne et al., 2017; Sakr, 2018).

When addressing the second research question regarding the influence of touchscreen use on young children’s social development, the research team systematically reviewed all relevant studies and conducted content analysis. The findings were categorized into three main categories: positive influences, challenges and considerations, and mediating factors. Touchscreen devices can positively impact young children’s social development in several ways. Multi-touch interfaces and tabletops facilitate collaborative learning by encouraging joint problem-solving and turn-taking, promoting social skills and cooperation (Jamil et al., 2017). Tablets and smartphones enhance peer interaction and collaboration as children work together to complete tasks, boosting social engagement and self-confidence. Video communication apps like Skype and Facetime on tablets support social play, allowing children to interact with family members and friends, thereby maintaining social bonds (Demirsu, 2020). In classroom settings, touchscreens, particularly iPads, foster increased peer collaboration, with children sharing knowledge, assisting each other, and engaging in group projects (Clarke and Abbott, 2016). Additionally, digital play with touchscreens encourages creative expression, as children share ideas and collaborate in activities like drawing and storytelling, promoting cooperation and social bonding.

However, there are also challenges associated with touchscreen use. Excessive screen time can reduce opportunities for face-to-face peer interactions and cooperation, as children may become more engrossed in individual screen time (Gou and Yang, 2024). Unsupervised use of technology can lead to passivity in social interactions and difficulties in peer relationships, potentially resulting in isolation. Single-user touchscreen displays can lead to parental disengagement, reducing opportunities for collaborative discussions and joint problem-solving, which are crucial for social development (Gülay Ogelman et al., 2016). Furthermore, touchscreen usage, particularly through electronic books, can negatively affect young children’s communication skills, as parents may focus more on the technology than the story content, displacing meaningful dialogue and reducing educationally enriching experiences (Munzer et al., 2019).

Several factors mediate the impact of touchscreen use on social development. Social scaffolding, where social demonstrations on touchscreen devices improve children’s ability to replicate actions, enhances understanding and task completion, fostering cooperation and joint attention. Individual differences also play a role, as collaboration among children is influenced more by personality traits than the method of interaction. Some children may dominate the use of the device, leading to withdrawal from others, while others may use it to assist one another. Cultural perceptions are also important, with parents expressing concerns that excessive time on touchscreens may limit opportunities for outdoor play and face-to-face interactions (Dardanou et al., 2020; Fotakopoulou et al., 2020). Finally, the presence and focus on iPads in early childhood settings can shape children’s interactions and behaviors, potentially reinforcing specific social behaviors and hierarchies.

To address the third research question regarding the influence of touchscreen use on young children’s communication skills, the research team systematically reviewed all selected studies and conducted content analysis. The findings were categorized into two main categories: positive influences and negative influences or challenges.

Touchscreen usage has been linked to several positive influences on children’s communication skills. Enhanced parent–child conversations were noted, as some apps encourage verbal interactions between children and parents. Touchscreens also facilitate collaborative play, promoting communication through cooperative activities. Interactive apps support language learning by helping with vocabulary building and storytelling, and some studies highlight how touchscreens can support bilingual or multilingual language development (Disney and Geng, 2022). Additionally, children who are typically quiet may feel more comfortable engaging in discussions when using touchscreens, thereby increasing their confidence.

However, there are also notable challenges associated with touchscreen use. Reduced verbal communication was observed, with children relying more on gestures instead of verbal interactions. Excessive screen time was linked to delays in expressive language and social communication. Parental distraction, where parents are absorbed in their own devices, can reduce verbal engagement with their children. Limited face-to-face interaction was another concern, as children using touchscreens alone may have fewer opportunities for conversational practice. Some studies also suggest that touchscreen use can make it harder for children to pick up social and emotional cues and decode social situations (Carson and Kuzik, 2021; Moser et al., 2015).

In response to the fourth research question regarding recommendations for touchscreen use for young children, the research team meticulously examined all studies and performed deductive content analysis with three pre-defined categories of recommendations: developmental, educational, and policy perspectives. Additionally, practical recommendations emerged from the data. Developmental recommendations emphasize balanced screen time, interactive engagement, guided use, and parental involvement to support overall development (Pasqualotto and Filosofi, 2023; Subiaul et al., 2015; Vartiainen et al., 2019). Researchers suggest limiting screen time and balancing it with traditional play and physical activities, encouraging co-viewing and co-playing with adults, and providing appropriate support during touchscreen use (Formby, 2014; Jamil et al., 2017; Morris et al., 2022; Sakr, 2018). Further empirical studies are needed to provide concrete guidelines for parents.

Educational recommendations focus on selecting high-quality educational apps, teacher facilitation, structured programs, collaborative learning, and teacher training. Reviewed studies recommend choosing apps that promote interaction and are educationally beneficial, actively facilitating touchscreen-based collaboration, and integrating digital tools into pedagogical strategies (Dashti and Habeeb, 2020; Kucirkova and Sakr, 2015). Structured programs in early childhood settings should promote hands-on learning experiences alongside digital activities, and tablet games should support collaborative learning by incorporating shared goals and meaningful interactions (Shalani et al., 2023; Yoo and Smetana, 2022). Teachers should receive training to effectively integrate technology into the classroom (Kubicki et al., 2015; Miller, 2018; Peña et al., 2024).

Policy recommendations include improving public education efforts, developing guidelines for app developers, encouraging screen-free family time, implementing supervised after-kindergarten programs, and educating parents on appropriate smart device usage (Hegedus and Otálora, 2023; Nacher et al., 2020; Quinones and Adams, 2021). Practical recommendations involve monitoring screen time, creating safe, engaging outdoor spaces, balancing educational content with traditional play, and considering digital screening tools for developmental assessments in pediatric clinics (Chaibal and Chaiyakul, 2022; Hashmi et al., 2022; Moon et al., 2019). These themes highlight the importance of a balanced approach to touchscreen use, integrating technology thoughtfully into educational practices, and providing clear guidelines and support for parents and educators (Marsh et al., 2020; Moser et al., 2015; Otterborn et al., 2019).

The systematic revealed that the impact of touchscreen technologies on children’s social development, communication, and collaboration is multifaceted and context-dependent. While digital tools are increasingly integrated into educational and domestic settings for their interactive and pedagogical potential, concerns remain regarding their overuse and the potential displacement of face-to-face interactions. Despite these concerns, a growing body of research underscores the enduring importance of social interaction in learning processes (Ren, 2023; Rocha and Nunes, 2020). When used thoughtfully, touchscreen technologies can support collaborative creativity, peer engagement, and family bonding, suggesting that the medium itself is not inherently detrimental but rather shaped by its usage context (Liu and Hwang, 2021).

A critical area that was also identified in the review concerns the contextual factors that mediate the effects of screen use, including parental involvement, peer dynamics, and the design of educational content. Emerging practices like “co-playing”—shared screen engagement between children and caregivers or peers—have shown promise in fostering communication and social learning (McArthur et al., 2022). This highlights the need for researchers to distinguish between solitary and socially mediated digital experiences, and to collaborate with developers and industry stakeholders in designing tools that promote children’s mental health, physical activity, and socio-emotional development (Christakis and Hale, 2025).

This review synthesized a broad spectrum of studies on touchscreen use among young children, examining device types, communication skills, and implications for practice and policy. The findings reveal both opportunities and challenges associated with the integration of touchscreen technologies into children’s daily routines and learning environments. Notably, the impact of touchscreen use is not uniform but is influenced by a constellation of social, contextual, and individual factors (Caballero-Julia et al., 2024).

The evidence supports the adoption of balanced, developmentally appropriate approaches to digital engagement in early childhood. Strategies such as co-use, guided participation, and policy frameworks that prioritize holistic child development are essential. In this regard, international human rights instruments provide valuable guidance. General Comment No. 20 (UN Committee on the Rights of the Child (UNCRC), 2016) emphasizes that the best interests of the child must be a primary consideration in all decisions affecting them, including those involving digital technologies. General Comment No. 25 (UN Committee on the Rights of the Child (UNCRC), 2021) further extends these rights into the digital realm, advocating for children’s meaningful participation in shaping their digital experiences.

This rights-based approach aligns with a growing international consensus that the development and governance of digital platforms must be informed by children’s developmental needs and vulnerabilities. It also reinforces the importance of distinguishing between different types of screen activities. For instance, co-viewing educational content or engaging in cooperative gameplay may yield developmental benefits, whereas solitary, passive, or overstimulating digital use may contribute to negative outcomes such as reduced social interaction, emotional dysregulation, or communication delays.

National initiatives, such as those led by the Norwegian Screen Committee (Norwegian Ministry of Education, 2024), underscore the importance of involving children in the creation of digital guidelines and interventions. These efforts demonstrate that trust, relevance, and compliance are enhanced when young people’s perspectives are integrated into public health messaging and digital literacy campaigns. Consequently, empirical research that centers children’ s lived experiences and perceptions is vital for the development of effective, equitable, and child-sensitive policies.

Lastly, protecting children’s well-being in the digital age requires interdisciplinary collaboration, ongoing research-informed policy development, and proactive efforts at the family, school, and governmental levels. These must be guided not only by evidence but also by an unwavering commitment to children’s rights and best interests in every aspect of their digital lives.

### 4.1 Limitations

This review found that touchscreens can facilitate children’s peer interaction in group activities, thereby enhancing social skills and communication development. However, there are still unanswered questions about how digital touchscreens contribute to delayed language development, basic communication skills (such as eye contact, face-to-face interaction and body language), and reduced parental engagement.

Additionally, our review focused only on studies involving typically developing children who, as the geographical distribution revealed, live mostly in Western societies. Consequently, little is known about how touchscreens may affect more diverse groups, such as neurodiverse children or those with other disabilities. Other sociocultural factors, such as age, ethnicity, and cultural capital, were largely overlooked in the reviewed studies (Torres et al., 2021). That suggests that meanings and aspects of social practices and communication skills vary significantly and could be influenced by other cultural contexts, potentially leading to different uses of touchscreens.

We encourage future research to explore these questions intentionally. Such studies could provide meaningful insights into the types of engagement and communication practices that different stakeholders, including teachers and parents, greatly benefit from in promoting effective early childhood education. Additionally, teacher education and professional development courses are equally important in supporting and equipping educators to develop developmentally appropriate pedagogical principles and digital competencies for effectively implementing touchscreens. This could enhance, thereby increasing authentic social development and communication skills. Finally, the review included only three databases. This may limit the comprehensiveness of the search and potentially exclude relevant studies published in other sources.

### 4.2 Conclusion

This research explores the complex impact of touchscreen devices on young children, examining various types such as tablets, smartphones, and interactive displays. It highlights how these technologies offer opportunities for learning and social interaction, especially with adult guidance and quality content. However, the impact of touchscreen technologies on young children’s social development, communication, and collaboration is highly nuanced and context dependent. The evidence suggests that these technologies are neither inherently beneficial nor detrimental; rather, their effects hinge on how, when, and with whom they are used. When touchscreen devices are integrated into children’s environments with thoughtful guidance, they can foster creativity, peer collaboration, and family engagement. Conversely, unsupervised, passive, or excessive use can displace critical real-world interactions, posing risks to emotional and communicative development.

One of the most prominent insights from literature is the need to distinguish between solitary screen use and socially mediated digital experiences. Co-use practices, such as co-playing and co-viewing, emerge as promising strategies that preserve the social function of learning while leveraging digital interactivity. These shared experiences can enhance communication skills, foster social bonds, and support the co-construction of knowledge. Adult mediation plays a crucial role in scaffolding these interactions and transforming digital use into a relational and developmental asset.

Considering these findings, policies and practices surrounding children’s digital engagement must move beyond blanket screen time limits and instead adopt a more research and developmentally informed framework. This includes recognizing the diverse ways children interact with technology and differentiating between content types, user intentions, and engagement contexts. Such an approach respects children’s evolving capacities and allows for the intentional design of digital environments that prioritize their well-being.

Moreover, there is growing recognition from an ecosystemic perspective of the importance of including children’s, educators’ and parents’ voices in shaping the digital spaces and their agency. It is on these three agents to be more participative in co-designing the tools and guidelines that affect them; only then are interventions more likely to be relevant, effective, and ethically grounded. This participatory ethos should extend to all levels of digital policy, education, and research.

Ultimately, the integration of touchscreen technologies into early childhood must be underpinned by a research and practice-based informed framework highly committed to children’s rights, inclusive principles, and adaptive digital environments. As digital media become ever more embedded in daily life, it is challenging for policy makers, researchers, educators and parents to work collaboratively, ensuring that digital tools and their content contribute meaningfully to children’s holistic development by nourishing children’s curiosity, social competence, and emotional resilience.

## Data Availability

The original contributions presented in the study are included in the article/Supplementary material, further inquiries can be directed to the corresponding authors.

## Footnotes

^1^ https://osf.io/9athz/?view_only=1fa731457d7e48248b7482bca02793f9
