# Supplementary material for: The impact of touchscreen digital exposure on children’s social development and communication: a systematic review
Source: Front Psychol. 2025 Oct 13;16:1613625. doi: 10.3389/fpsyg.2025.1613625 (PMC12557575; doi:10.3389/fpsyg.2025.1613625)
Supplement: Supplementary file 1 [file Data_Sheet_1.pdf]

Appendix A: Search strings and queries

Database	String	Query
**WoS**	"smartphone*" AND "child*" AND "social"	((AB=((((((((((((((((AB=("smartphone*")) AND AB=("child*")) AND AB=("social")) NOT AB=("disorder")) NOT AB=("autism")) NOT AB=("autistic")) NOT AB=("spectrum")) NOT AB=("anxiety")) NOT AB=("depression")) NOT AB=("disability")) NOT AB=("Parkinson")) NOT AB=("adolescent*")) NOT AB=("addiction")) NOT AB=("disadvantages")) NOT AB=("delay")) NOT AB=("elderly")) NOT AB=("elementary")) NOT AB=("media"))) NOT AB=("Youth")) NOT AB=("older")
	"smartphone*" AND "child*" AND "communication"	((((((AB=("smartphone*")) AND AB=("child")) AND AB=("communication")) NOT AB=("autism")) NOT AB=("disorder")) NOT AB=("health")) NOT ALL=("Adolescents")) NOT AB=("tracheostomy")
	"smartphone*" AND "child*" AND "collaboration"	((((((AB=("smartphone*")) AND AB=("child")) AND AB=("collabotation")) NOT AB=("autism")) NOT AB=("disorder")) NOT AB=("health")) NOT ALL=("Adolescents")) NOT AB=("tracheostomy")
	"tablet*" AND "child*" AND "social"	(((((((AB=("tablet*")) AND AB=("child")) AND AB=("social")) NOT AB=("autism")) NOT AB=("disorder*")) NOT ALL=("Adolescent*")) NOT AB=("Stigma")) NOT AB=("feeding")) NOT AB=("health")
	"tablet*" AND "child*" AND "communication"	(((((((AB=("tablet*")) AND AB=("child")) AND AB=("communication")) NOT AB=("autism")) NOT AB=("disorder*")) NOT ALL=("Adolescent*")) NOT AB=("Stigma")) NOT AB=("feeding")) NOT AB=("health")
	"tablet*" AND "child*" AND "collaboration"	((((((((((((((AB=("tablet*")) AND AB=("child")) AND AB=("collaboration")) NOT AB=("autism")) NOT AB=("disorder")) NOT AB=("autistic")) NOT AB=("health")) NOT AB=("primary")) NOT AB=("secondary")) NOT AB=("ADHD")) NOT AB=("physical phenomena ")) NOT AB=("primary school")) NOT AB=("pediatric")) NOT AB=("grammar school")) NOT AB=("Nearpod application")) NOT AB=("digital eye")) NOT AB=("clinical")
	"ipad*" AND "child*" AND "social"	(((((((((AB=("iPad*")) AND AB=("child")) AND AB=("social")) NOT AB=("autism")) NOT AB=("disorder")) NOT AB=("health")) NOT AB=("autistic")) NOT AB=("deaf")) NOT AB=("pathologist")) NOT AB=("cerebral palsy")) NOT AB=("clinical")
	"ipad*" AND "child*" AND "communication"	(((((((((((AB=("Ipad*")) AND AB=("child")) AND AB=("communication")) NOT ALL=("disorder*")) NOT AB=("autism")) NOT AB=("adolescent*")) NOT AB=("clinical*")) NOT AB=("vocabulary")) NOT AB=("writing")) NOT AB=("special")) NOT AB=("deaf")) NOT AB=("telemedicine")) NOT AB=("disability")
	"ipad*" AND "child*" AND "collaboration"	((((AB=("iPad*")) AND AB=("child")) AND AB=("collaboration")) NOT AB=("autism")) NOT AB=("disorder*")
**ERIC**	"smartphone*" AND "child*" AND "social"	

	"smartphone*" AND "child*" AND "communication"	
	"smartphone*" AND "child*" AND "collaboration"	
	"tablet*" AND "child*" AND "social"	
	"tablet*" AND "child*" AND "communication"	
	"tablet*" AND "child*" AND "collaboration"	
	"ipad*" AND "child*" AND "social"	
	"ipad*" AND "child*" AND "communication"	
	"ipad*" AND "child*" AND "collaboration"	
**SCOPUS**	"smartphone*" AND "child*" AND "social"	(TITLE-ABS-KEY ("smartphone*") AND TITLE-ABS-KEY ("child*") AND TITLE-ABS-KEY ("social") AND NOT TITLE-ABS-KEY ("youth") OR TITLE-ABS-KEY ("adolescent*") OR TITLE-ABS-KEY ("autism")) AND PUBYEAR > 2013 AND PUBYEAR < 2025 AND (EXCLUDE (SUBJAREA , "MEDI") OR EXCLUDE (SUBJAREA , "ENGI") OR EXCLUDE (SUBJAREA , "NURS") OR EXCLUDE (SUBJAREA , "ENVI") OR EXCLUDE (SUBJAREA , "NEUR") OR EXCLUDE (SUBJAREA , "MULT") OR EXCLUDE (SUBJAREA , "HEAL") OR EXCLUDE (SUBJAREA , "MATH") OR EXCLUDE (SUBJAREA , "BUSI") OR EXCLUDE (SUBJAREA , "DECI") OR EXCLUDE (SUBJAREA , "BIOC") OR EXCLUDE (SUBJAREA , "PHAR") OR EXCLUDE (SUBJAREA , "AGRI") OR EXCLUDE (SUBJAREA , "PHYS") OR EXCLUDE (SUBJAREA , "IMMU") OR EXCLUDE (SUBJAREA , "ENER") OR EXCLUDE (SUBJAREA , "ECON") OR EXCLUDE (SUBJAREA , "CENG") OR EXCLUDE (SUBJAREA , "MATE") OR EXCLUDE (SUBJAREA , "EART") OR EXCLUDE (SUBJAREA , "DENT") OR EXCLUDE (SUBJAREA , "CHEM")) AND (EXCLUDE (DOCTYPE , "cp") OR EXCLUDE (DOCTYPE , "ch") OR EXCLUDE (DOCTYPE , "cr") OR EXCLUDE (DOCTYPE , "bk")) AND (EXCLUDE (LANGUAGE , "Spanish") OR EXCLUDE (LANGUAGE , "Russian") OR EXCLUDE (LANGUAGE , "Afrikaans") OR EXCLUDE (LANGUAGE , "Malay") OR EXCLUDE (LANGUAGE , "Italian") OR EXCLUDE (LANGUAGE , "Portuguese") OR EXCLUDE (LANGUAGE , "Chinese")) AND (EXCLUDE (EXACTKEYWORD , "Advertising") OR EXCLUDE (EXACTKEYWORD , "Information Processing") OR EXCLUDE (EXACTKEYWORD , "Middle Aged") OR EXCLUDE (EXACTKEYWORD , "Mental Stress") OR EXCLUDE (EXACTKEYWORD , "Ownership") OR EXCLUDE (EXACTKEYWORD , "Personal Satisfaction") OR EXCLUDE (EXACTKEYWORD , "Personal Experience") OR EXCLUDE (EXACTKEYWORD , "Ecological Momentary Assessment") OR EXCLUDE (EXACTKEYWORD , "Facebook") OR EXCLUDE (

		EXACTKEYWORD , "Internet Gaming Disorder") OR EXCLUDE (EXACTKEYWORD , "MHealth") OR EXCLUDE (EXACTKEYWORD , "Mindfulness") OR EXCLUDE (EXACTKEYWORD , "Phubbing") OR EXCLUDE (EXACTKEYWORD , "Physiology") OR EXCLUDE (EXACTKEYWORD , "Reproducibility") OR EXCLUDE (EXACTKEYWORD , "Reproducibility Of Results") OR EXCLUDE (EXACTKEYWORD , "Sex Factors") OR EXCLUDE (EXACTKEYWORD , "Social Networking (online)") OR EXCLUDE (EXACTKEYWORD , "Social Networking Sites") OR EXCLUDE (EXACTKEYWORD , "Wellbeing") OR EXCLUDE (EXACTKEYWORD , "Behavior, Addictive") OR EXCLUDE (EXACTKEYWORD , "Distress Syndrome") OR EXCLUDE (EXACTKEYWORD , "Gender") OR EXCLUDE (EXACTKEYWORD , "Social Media Addiction") OR EXCLUDE (EXACTKEYWORD , "Addiction") OR EXCLUDE (EXACTKEYWORD , "Adolescence") OR EXCLUDE (EXACTKEYWORD , "Anxiety") OR EXCLUDE (EXACTKEYWORD , "Adolescent Behavior") OR EXCLUDE (EXACTKEYWORD , "COVID-19") OR EXCLUDE (EXACTKEYWORD , "Depression") OR EXCLUDE (EXACTKEYWORD , "Internet Addiction") OR EXCLUDE (EXACTKEYWORD , "Mental Health") OR EXCLUDE (EXACTKEYWORD , "Technoference") OR EXCLUDE (EXACTKEYWORD , "Adolescents") OR EXCLUDE (EXACTKEYWORD , "Adolescent") OR EXCLUDE (EXACTKEYWORD , "Humans") OR EXCLUDE (EXACTKEYWORD , "Human") OR EXCLUDE (EXACTKEYWORD , "Female") OR EXCLUDE (EXACTKEYWORD , "Male") OR EXCLUDE (EXACTKEYWORD , "Social Media") OR EXCLUDE (EXACTKEYWORD , "-Cybersecurity") OR EXCLUDE (EXACTKEYWORD , "-cybersecurity") OR EXCLUDE (EXACTKEYWORD , "ADHD Symptoms") OR EXCLUDE (EXACTKEYWORD , "Adolescent Reading") OR EXCLUDE (EXACTKEYWORD , "Adolescents And Young Adults") OR EXCLUDE (EXACTKEYWORD , "Age Classification") OR EXCLUDE (EXACTKEYWORD , "Anthropocene") OR EXCLUDE (EXACTKEYWORD , "Attention Deficit Hyperactivity Disorder (ADHD)") OR EXCLUDE (EXACTKEYWORD , "Audience Measurement") OR EXCLUDE (EXACTKEYWORD , "Bagging And Boosting") OR EXCLUDE (EXACTKEYWORD , "Behavioral Problems") OR EXCLUDE (EXACTKEYWORD , "Bidirectionality") OR EXCLUDE (EXACTKEYWORD , "Boss Phubbing") OR EXCLUDE (EXACTKEYWORD , "COVID-19 Pandemic") OR EXCLUDE (EXACTKEYWORD , "Care") OR EXCLUDE (EXACTKEYWORD , "Cartography") OR EXCLUDE (EXACTKEYWORD , "Childhood Emotional Neglect") OR EXCLUDE (EXACTKEYWORD , "Children Heading Households") OR EXCLUDE (EXACTKEYWORD , "Children Phubbing") OR EXCLUDE (EXACTKEYWORD , "Color Characteristics") OR EXCLUDE (EXACTKEYWORD , "Community Mental Health") OR EXCLUDE (EXACTKEYWORD , "Computational Framework") OR EXCLUDE (EXACTKEYWORD , "Computational Infrastructure") OR EXCLUDE (

		EXACTKEYWORD , "Computer Networks") OR EXCLUDE (EXACTKEYWORD , "Coping Using Media") OR EXCLUDE (EXACTKEYWORD , "Control Engineering") OR EXCLUDE (EXACTKEYWORD , "Covid-19") OR EXCLUDE (EXACTKEYWORD , "Crib Song") OR EXCLUDE (EXACTKEYWORD , "Cross-cultural Adjustment") OR EXCLUDE (EXACTKEYWORD , "Crowds") OR EXCLUDE (EXACTKEYWORD , "Cryptography") OR EXCLUDE (EXACTKEYWORD , "Cyber Security") OR EXCLUDE (EXACTKEYWORD , "Cybersecurity") OR EXCLUDE (EXACTKEYWORD , "Disaster Simulation") OR EXCLUDE (EXACTKEYWORD , "Displacement") OR EXCLUDE (EXACTKEYWORD , "Distracted Parenting"))
	"smartphone*" AND "child*" AND "communication"	(TITLE-ABS-KEY ("smartphone*") AND TITLE-ABS-KEY ("child*") AND TITLE-ABS-KEY ("communication") AND NOT TITLE-ABS-KEY ("youth") OR TITLE-ABS-KEY ("adolescents") OR TITLE-ABS-KEY ("autism")) AND PUBYEAR > 2013 AND PUBYEAR < 2024 AND (EXCLUDE (SUBJAREA , "MEDI") OR EXCLUDE (SUBJAREA , "ENGI") OR EXCLUDE (SUBJAREA , "MATH") OR EXCLUDE (SUBJAREA , "NURS") OR EXCLUDE (SUBJAREA , "HEAL") OR EXCLUDE (SUBJAREA , "ENVI") OR EXCLUDE (SUBJAREA , "DECI") OR EXCLUDE (SUBJAREA , "PHYS") OR EXCLUDE (SUBJAREA , "BIOC") OR EXCLUDE (SUBJAREA , "MATE") OR EXCLUDE (SUBJAREA , "NEUR") OR EXCLUDE (SUBJAREA , "ENER") OR EXCLUDE (SUBJAREA , "MULT") OR EXCLUDE (SUBJAREA , "IMMU") OR EXCLUDE (SUBJAREA , "BUSI") OR EXCLUDE (SUBJAREA , "CENG") OR EXCLUDE (SUBJAREA , "PHAR") OR EXCLUDE (SUBJAREA , "ECON") OR EXCLUDE (SUBJAREA , "EART") OR EXCLUDE (SUBJAREA , "CHEM") OR EXCLUDE (SUBJAREA , "AGRI") OR EXCLUDE (SUBJAREA , "DENT")) AND (EXCLUDE (DOCTYPE , "cp") OR EXCLUDE (DOCTYPE , "ch") OR EXCLUDE (DOCTYPE , "cr") OR EXCLUDE (DOCTYPE , "bk")) AND (LIMIT-TO (LANGUAGE , "English")) AND (EXCLUDE (EXACTKEYWORD , "APPs") OR EXCLUDE (EXACTKEYWORD , "ATIS") OR EXCLUDE (EXACTKEYWORD , "Accessibility") OR EXCLUDE (EXACTKEYWORD , "Adaptation") OR EXCLUDE (EXACTKEYWORD , "Adaptation, Psychological") OR EXCLUDE (EXACTKEYWORD , "Agency") OR EXCLUDE (EXACTKEYWORD , "Aggression") OR EXCLUDE (EXACTKEYWORD , "Ambulatory Assessment") OR EXCLUDE (EXACTKEYWORD , "Anxiety") OR EXCLUDE (EXACTKEYWORD , "Application Programs") OR EXCLUDE (EXACTKEYWORD , "Apps") OR EXCLUDE (EXACTKEYWORD , "Android Apps") OR EXCLUDE (EXACTKEYWORD , "Android (operating System)") OR EXCLUDE (EXACTKEYWORD , "Area Of Interest") OR EXCLUDE (EXACTKEYWORD , "Art-based Methods") OR EXCLUDE (EXACTKEYWORD , "Arts-based Methods") OR EXCLUDE (EXACTKEYWORD , "At-risk Young Children") OR EXCLUDE (EXACTKEYWORD , "Augmented Reality") OR EXCLUDE (EXACTKEYWORD , "Attention Disturbance") OR EXCLUDE (

		EXACTKEYWORD , "Authentication") OR EXCLUDE (EXACTKEYWORD , "Automatic Content") OR EXCLUDE (EXACTKEYWORD , "Autonomous Cars") OR EXCLUDE (EXACTKEYWORD , "Bangladesh") OR EXCLUDE (EXACTKEYWORD , "Behavior Therapy") OR EXCLUDE (EXACTKEYWORD , "Behavioral Parent Training") OR EXCLUDE (EXACTKEYWORD , "Behavioral Problems") OR EXCLUDE (EXACTKEYWORD , "Bi-factor Analysis") OR EXCLUDE (EXACTKEYWORD , "Bidirectionality") OR EXCLUDE (EXACTKEYWORD , "Biological Features") OR EXCLUDE (EXACTKEYWORD , "Biometric Verification") OR EXCLUDE (EXACTKEYWORD , "Biometrics") OR EXCLUDE (EXACTKEYWORD , "Birth Parents") OR EXCLUDE (EXACTKEYWORD , "Bluetooth Connections") OR EXCLUDE (EXACTKEYWORD , "Book") OR EXCLUDE (EXACTKEYWORD , "Brand Communication") OR EXCLUDE (EXACTKEYWORD , "Books") OR EXCLUDE (EXACTKEYWORD , "Cartography") OR EXCLUDE (EXACTKEYWORD , "Caregivers") OR EXCLUDE (EXACTKEYWORD , "CMC") OR EXCLUDE (EXACTKEYWORD , "CCTV") OR EXCLUDE (EXACTKEYWORD , "Cellular Telephone Systems") OR EXCLUDE (EXACTKEYWORD , "Cellular Telephones") OR EXCLUDE (EXACTKEYWORD , "Child Behavior Checklist") OR EXCLUDE (EXACTKEYWORD , "Child Care Center") OR EXCLUDE (EXACTKEYWORD , "Child Monitoring") OR EXCLUDE (EXACTKEYWORD , "Child-effect") OR EXCLUDE (EXACTKEYWORD , "Children Android Applications (Apps)") OR EXCLUDE (EXACTKEYWORD , "Children Online") OR EXCLUDE (EXACTKEYWORD , "Children's Rights") OR EXCLUDE (EXACTKEYWORD , "Chronic Disease") OR EXCLUDE (EXACTKEYWORD , "Chronic Diseases") OR EXCLUDE (EXACTKEYWORD , "Clinical Effectiveness") OR EXCLUDE (EXACTKEYWORD , "Clinical Practice") OR EXCLUDE (EXACTKEYWORD , "Cloud Computing") OR EXCLUDE (EXACTKEYWORD , "Cloud Servers") OR EXCLUDE (EXACTKEYWORD , "Co-making") OR EXCLUDE (EXACTKEYWORD , "Cognition Assessment") OR EXCLUDE (EXACTKEYWORD , "Communication Network") OR EXCLUDE (EXACTKEYWORD , "Computer Forensics") OR EXCLUDE (EXACTKEYWORD , "Computer Addiction") OR EXCLUDE (EXACTKEYWORD , "Communications Effects") OR EXCLUDE (EXACTKEYWORD , "Communication Technologies") OR EXCLUDE (EXACTKEYWORD , "Computer Games") OR EXCLUDE (EXACTKEYWORD , "Computer Networks") OR EXCLUDE (EXACTKEYWORD , "Computers, Handheld") OR EXCLUDE (EXACTKEYWORD , "Constructionist Learning") OR EXCLUDE (EXACTKEYWORD , "Contact") OR EXCLUDE (EXACTKEYWORD , "Content Forensics") OR EXCLUDE (EXACTKEYWORD , "Content Inspection") OR EXCLUDE (EXACTKEYWORD , "Continuous Monitoring") OR EXCLUDE (EXACTKEYWORD , "Control Software") OR EXCLUDE (EXACTKEYWORD , "Control") OR EXCLUDE (EXACTKEYWORD , "Controlled Study") OR EXCLUDE (EXACTKEYWORD , "Coping Behavior") OR EXCLUDE (EXACTKEYWORD , "Cost Effectiveness") OR EXCLUDE

		(EXACTKEYWORD , "Data Privacy") OR EXCLUDE (EXACTKEYWORD , "Data Fusion") OR EXCLUDE (EXACTKEYWORD , "Cross-sectional Surveys") OR EXCLUDE (EXACTKEYWORD , "Cross-sectional Study") OR EXCLUDE (EXACTKEYWORD , "Croatia") OR EXCLUDE (EXACTKEYWORD , "Database- Driven Applications") OR EXCLUDE (EXACTKEYWORD , "Decision Support System (DSS)") OR EXCLUDE (EXACTKEYWORD , "Design") OR EXCLUDE (EXACTKEYWORD , "Depression") OR EXCLUDE (EXACTKEYWORD , "Dependable Systems") OR EXCLUDE (EXACTKEYWORD , "Demography") OR EXCLUDE (EXACTKEYWORD , "Design And Evaluations") OR EXCLUDE (EXACTKEYWORD , "Design And Implementations") OR EXCLUDE (EXACTKEYWORD , "Digital") OR EXCLUDE (EXACTKEYWORD , "Difficulty") OR EXCLUDE (EXACTKEYWORD , "Dialectical Behavioral Therapy") OR EXCLUDE (EXACTKEYWORD , "Developmental Language Disorder") OR EXCLUDE (EXACTKEYWORD , "Design/methodology/approach") OR EXCLUDE (EXACTKEYWORD , "Digital Government") OR EXCLUDE (EXACTKEYWORD , "Digital Parenting") OR EXCLUDE (EXACTKEYWORD , "Digitization") OR EXCLUDE (EXACTKEYWORD , "Disaster Simulation") OR EXCLUDE (EXACTKEYWORD , "Diseases") OR EXCLUDE (EXACTKEYWORD , "EHealth") OR EXCLUDE (EXACTKEYWORD , "Communications Media") OR EXCLUDE (EXACTKEYWORD , "Child Behavior") OR EXCLUDE (EXACTKEYWORD , "Caregiver") OR EXCLUDE (EXACTKEYWORD , "Face Recognition") OR EXCLUDE (EXACTKEYWORD , "Gender") OR EXCLUDE (EXACTKEYWORD , "GPS") OR EXCLUDE (EXACTKEYWORD , "Global System For Mobile Communications") OR EXCLUDE (EXACTKEYWORD , "Mass Medium") OR EXCLUDE (EXACTKEYWORD , "Mobile Application") OR EXCLUDE (EXACTKEYWORD , "Location") OR EXCLUDE (EXACTKEYWORD , "Mobile Phones") OR EXCLUDE (EXACTKEYWORD , "Mobile Telecommunication Systems") OR EXCLUDE (EXACTKEYWORD , "Motivation") OR EXCLUDE (EXACTKEYWORD , "Negotiating") OR EXCLUDE (EXACTKEYWORD , "Parent-Child Relations") OR EXCLUDE (EXACTKEYWORD , "Perception") OR EXCLUDE (EXACTKEYWORD , "Priority Journal") OR EXCLUDE (EXACTKEYWORD , "Photography") OR EXCLUDE (EXACTKEYWORD , "Problem Behavior") OR EXCLUDE (EXACTKEYWORD , "Semantics") OR EXCLUDE (EXACTKEYWORD , "Wearable Sensors") OR EXCLUDE (EXACTKEYWORD , "Twitter") OR EXCLUDE (EXACTKEYWORD , "Social Media") OR EXCLUDE (EXACTKEYWORD , "WhatsApp") OR EXCLUDE (EXACTKEYWORD , "Addiction") OR EXCLUDE (EXACTKEYWORD , "Adult") OR EXCLUDE (EXACTKEYWORD , "COVID-19") OR EXCLUDE (EXACTKEYWORD , "Facebook") OR EXCLUDE (EXACTKEYWORD , "Internet Of Things") OR EXCLUDE (EXACTKEYWORD , "Internet") OR EXCLUDE (EXACTKEYWORD , "Family Communication") OR EXCLUDE (EXACTKEYWORD , "Parenting") OR

		EXCLUDE (EXACTKEYWORD , "Child Parent Relation") OR EXCLUDE (EXACTKEYWORD , "Questionnaire") OR EXCLUDE (EXACTKEYWORD , "Surveys And Questionnaires") OR EXCLUDE (EXACTKEYWORD , "Female") OR EXCLUDE (EXACTKEYWORD , "Human") OR EXCLUDE (EXACTKEYWORD , "Male") OR EXCLUDE (EXACTKEYWORD , "Humans"))
	"smartphone*" AND "child*" AND "collaboration"	(TITLE-ABS-KEY ("smartphone*") AND TITLE-ABS-KEY ("child*") AND TITLE-ABS-KEY ("collaboration") AND NOT TITLE-ABS-KEY ("youth") OR TITLE-ABS-KEY ("adolescents") OR TITLE-ABS-KEY ("autism")) AND PUBYEAR > 2013 AND PUBYEAR < 2024 AND (EXCLUDE (SUBJAREA , "MEDI") OR EXCLUDE (SUBJAREA , "ENGI") OR EXCLUDE (SUBJAREA , "MATH") OR EXCLUDE (SUBJAREA , "NURS") OR EXCLUDE (SUBJAREA , "HEAL") OR EXCLUDE (SUBJAREA , "ENVI") OR EXCLUDE (SUBJAREA , "DECI") OR EXCLUDE (SUBJAREA , "PHYS") OR EXCLUDE (SUBJAREA , "BIOC") OR EXCLUDE (SUBJAREA , "MATE") OR EXCLUDE (SUBJAREA , "NEUR") OR EXCLUDE (SUBJAREA , "ENER") OR EXCLUDE (SUBJAREA , "MULT") OR EXCLUDE (SUBJAREA , "IMMU") OR EXCLUDE (SUBJAREA , "BUSI") OR EXCLUDE (SUBJAREA , "CENG") OR EXCLUDE (SUBJAREA , "PHAR") OR EXCLUDE (SUBJAREA , "ECON") OR EXCLUDE (SUBJAREA , "EART") OR EXCLUDE (SUBJAREA , "CHEM") OR EXCLUDE (SUBJAREA , "AGRI") OR EXCLUDE (SUBJAREA , "DENT")) AND (EXCLUDE (DOCTYPE , "cp") OR EXCLUDE (DOCTYPE , "ch") OR EXCLUDE (DOCTYPE , "cr") OR EXCLUDE (DOCTYPE , "bk")) AND (LIMIT-TO (LANGUAGE , "English"))
	"tablet*" AND "child*" AND "social"	(TITLE-ABS-KEY ("tablet*") AND TITLE-ABS-KEY ("child*") AND TITLE-ABS-KEY ("social") AND NOT TITLE-ABS-KEY ("youth") OR TITLE-ABS-KEY ("adolescents") OR TITLE-ABS-KEY ("autism")) AND PUBYEAR > 2013 AND PUBYEAR < 2025 AND (EXCLUDE (SUBJAREA , "MEDI") OR EXCLUDE (SUBJAREA , "ENGI") OR EXCLUDE (SUBJAREA , "MATH") OR EXCLUDE (SUBJAREA , "NURS") OR EXCLUDE (SUBJAREA , "PHAR") OR EXCLUDE (SUBJAREA , "HEAL") OR EXCLUDE (SUBJAREA , "MULT") OR EXCLUDE (SUBJAREA , "ENVI") OR EXCLUDE (SUBJAREA , "BUSI") OR EXCLUDE (SUBJAREA , "AGRI") OR EXCLUDE (SUBJAREA , "PHYS") OR EXCLUDE (SUBJAREA , "DECI") OR EXCLUDE (SUBJAREA , "BIOC") OR EXCLUDE (SUBJAREA , "MATE") OR EXCLUDE (SUBJAREA , "ECON") OR EXCLUDE (SUBJAREA , "DENT") OR EXCLUDE (SUBJAREA , "IMMU") OR EXCLUDE (SUBJAREA , "ENER") OR EXCLUDE (SUBJAREA , "CHEM") OR EXCLUDE (SUBJAREA , "VETE") OR EXCLUDE (SUBJAREA , "CENG") OR EXCLUDE (SUBJAREA , "NEUR")) AND (EXCLUDE (DOCTYPE , "cp") OR EXCLUDE (DOCTYPE , "ch") OR EXCLUDE (DOCTYPE , "cr") OR EXCLUDE (DOCTYPE , "bk")) AND (EXCLUDE (LANGUAGE , "Afrikaans") OR EXCLUDE (LANGUAGE , "Russian") OR EXCLUDE (LANGUAGE , "Portuguese") OR EXCLUDE (LANGUAGE , "Chinese")) AND (EXCLUDE (

		EXACTKEYWORD , "Human") OR EXCLUDE (EXACTKEYWORD , "Humans") OR EXCLUDE (EXACTKEYWORD , "Economic And Social Effects") OR EXCLUDE (EXACTKEYWORD , "Female") OR EXCLUDE (EXACTKEYWORD , "Male") OR EXCLUDE (EXACTKEYWORD , "Literacy") OR EXCLUDE (EXACTKEYWORD , "Human-robot Interaction") OR EXCLUDE (EXACTKEYWORD , "E-learning") OR EXCLUDE (EXACTKEYWORD , "Communication") OR EXCLUDE (EXACTKEYWORD , "Collaborative Learning") OR EXCLUDE (EXACTKEYWORD , "Cognitive Development") OR EXCLUDE (EXACTKEYWORD , "Behavioral Research") OR EXCLUDE (EXACTKEYWORD , "Attention") OR EXCLUDE (EXACTKEYWORD , "Technology Addiction") OR EXCLUDE (EXACTKEYWORD , "Rural Area") OR EXCLUDE (EXACTKEYWORD , "Research Questions") OR EXCLUDE (EXACTKEYWORD , "Older Adults") OR EXCLUDE (EXACTKEYWORD , "Media Use") OR EXCLUDE (EXACTKEYWORD , "Language Learning") OR EXCLUDE (EXACTKEYWORD , "Imitation") OR EXCLUDE (EXACTKEYWORD , "Hand Held Computers") OR EXCLUDE (EXACTKEYWORD , "Cross-sectional Study") OR EXCLUDE (EXACTKEYWORD , "Child Development") OR EXCLUDE (EXACTKEYWORD , "AR System") OR EXCLUDE (EXACTKEYWORD , "ASR-based Translator") OR EXCLUDE (EXACTKEYWORD , "Abu Dhabi") OR EXCLUDE (EXACTKEYWORD , "Academic Achievement") OR EXCLUDE (EXACTKEYWORD , "Activity Photos") OR EXCLUDE (EXACTKEYWORD , "Acceptance") OR EXCLUDE (EXACTKEYWORD , "Academic Competencies") OR EXCLUDE (EXACTKEYWORD , "Addiction") OR EXCLUDE (EXACTKEYWORD , "Adoption") OR EXCLUDE (EXACTKEYWORD , "Adversarial Machine Learning") OR EXCLUDE (EXACTKEYWORD , "Age Classification") OR EXCLUDE (EXACTKEYWORD , "Applications In Coding") OR EXCLUDE (EXACTKEYWORD , "Application In Coding") OR EXCLUDE (EXACTKEYWORD , "Article") OR EXCLUDE (EXACTKEYWORD , "Artifact") OR EXCLUDE (EXACTKEYWORD , "Associated Sets") OR EXCLUDE (EXACTKEYWORD , "Attendance") OR EXCLUDE (EXACTKEYWORD , "Audience Measurement") OR EXCLUDE (EXACTKEYWORD , "Behavior Assessment") OR EXCLUDE (EXACTKEYWORD , "Behavior Rating Scale") OR EXCLUDE (EXACTKEYWORD , "Behavior, Addictive") OR EXCLUDE (EXACTKEYWORD , "Belgium") OR EXCLUDE (EXACTKEYWORD , "Bidirectionality") OR EXCLUDE (EXACTKEYWORD , "Board Games") OR EXCLUDE (EXACTKEYWORD , "Books In The Home") OR EXCLUDE (EXACTKEYWORD , "Bodies") OR EXCLUDE (EXACTKEYWORD , "Bridge-knowledge") OR EXCLUDE (EXACTKEYWORD , "COVID- 19") OR EXCLUDE (EXACTKEYWORD , "COVID-19") OR EXCLUDE (EXACTKEYWORD , "Caregivers") OR EXCLUDE (EXACTKEYWORD , "Children With Disabilities") OR EXCLUDE (EXACTKEYWORD , "Children With Developmental Delays") OR EXCLUDE (

		EXACTKEYWORD , "Children With ADHD") OR EXCLUDE (EXACTKEYWORD , "Children Scale") OR EXCLUDE (EXACTKEYWORD , "Child-robot Interaction") OR EXCLUDE (EXACTKEYWORD , "Child-effect") OR EXCLUDE (EXACTKEYWORD , "Child's Drawing") OR EXCLUDE (EXACTKEYWORD , "Child With ADHD") OR EXCLUDE (EXACTKEYWORD , "Child Protection") OR EXCLUDE (EXACTKEYWORD , "Child Museum") OR EXCLUDE (EXACTKEYWORD , "Cerebrovascular Accident") OR EXCLUDE (EXACTKEYWORD , "Ceaseless Parenting") OR EXCLUDE (EXACTKEYWORD , "Categorization") OR EXCLUDE (EXACTKEYWORD , "Children's Spirituality") OR EXCLUDE (EXACTKEYWORD , "Children's Museum") OR EXCLUDE (EXACTKEYWORD , "Children's Drawings") OR EXCLUDE (EXACTKEYWORD , "Color Characteristics") OR EXCLUDE (EXACTKEYWORD , "Collaboration") OR EXCLUDE (EXACTKEYWORD , "Cognitive Learning Theory") OR EXCLUDE (EXACTKEYWORD , "Cognitive Dysfunction") OR EXCLUDE (EXACTKEYWORD , "Cognitive Defect") OR EXCLUDE (EXACTKEYWORD , "Coding Skills") OR EXCLUDE (EXACTKEYWORD , "Co-viewing") OR EXCLUDE (EXACTKEYWORD , "Co-creation") OR EXCLUDE (EXACTKEYWORD , "Cloud Computing") OR EXCLUDE (EXACTKEYWORD , "Clinical Article") OR EXCLUDE (EXACTKEYWORD , "Chronic Illness") OR EXCLUDE (EXACTKEYWORD , "Children's Viewing") OR EXCLUDE (EXACTKEYWORD , "Content Discoverability") OR EXCLUDE (EXACTKEYWORD , "Connected Screens") OR EXCLUDE (EXACTKEYWORD , "Condition") OR EXCLUDE (EXACTKEYWORD , "Concept Development") OR EXCLUDE (EXACTKEYWORD , "Computers In Education") OR EXCLUDE (EXACTKEYWORD , "Computer-assisted Language Learning") OR EXCLUDE (EXACTKEYWORD , "Computer Supported Learning Environments") OR EXCLUDE (EXACTKEYWORD , "Computer Interface") OR EXCLUDE (EXACTKEYWORD , "Computer Aided Instruction") OR EXCLUDE (EXACTKEYWORD , "Computer Addiction") OR EXCLUDE (EXACTKEYWORD , "Computer") OR EXCLUDE (EXACTKEYWORD , "'current") OR EXCLUDE (EXACTKEYWORD , "Copying Behavior") OR EXCLUDE (EXACTKEYWORD , "Cooperative/collaborative Learning") OR EXCLUDE (EXACTKEYWORD , "Cooperative/ Collaborative Learning") OR EXCLUDE (EXACTKEYWORD , "Controlled Study") OR EXCLUDE (EXACTKEYWORD , "Cooperative Gestures") OR EXCLUDE (EXACTKEYWORD , "Contrastive Learning") OR EXCLUDE (EXACTKEYWORD , "Inhibitory Control") OR EXCLUDE (EXACTKEYWORD , "Ants") OR EXCLUDE (EXACTKEYWORD , "Coding Robots") OR EXCLUDE (EXACTKEYWORD , "Coding Apps") OR EXCLUDE (EXACTKEYWORD , "Computational Thinking") OR EXCLUDE (EXACTKEYWORD , "Photograph Album") OR EXCLUDE (EXACTKEYWORD , "Phenomenology") OR EXCLUDE (EXACTKEYWORD , "Population Geography") OR EXCLUDE (EXACTKEYWORD , "Preadolescents") OR EXCLUDE (

		EXACTKEYWORD , "Preadolescent") OR EXCLUDE (EXACTKEYWORD , "Rapid Prototyping") OR EXCLUDE (EXACTKEYWORD , "Religion") OR EXCLUDE (EXACTKEYWORD , "Self-disclosure") OR EXCLUDE (EXACTKEYWORD , "Self-Disclosure") OR EXCLUDE (EXACTKEYWORD , "Skype"))
	"tablet*" AND "child*" AND "communication"	(TITLE-ABS-KEY ("tablet*") AND TITLE-ABS-KEY ("child*") AND TITLE-ABS-KEY ("communication") AND NOT TITLE-ABS-KEY ("youth") OR TITLE-ABS-KEY ("adolescence") OR TITLE-ABS-KEY ("autism")) AND PUBYEAR > 2013 AND PUBYEAR < 2025 AND (EXCLUDE (SUBJAREA , "MEDI") OR EXCLUDE (SUBJAREA , "ENGI") OR EXCLUDE (SUBJAREA , "HEAL") OR EXCLUDE (SUBJAREA , "NURS") OR EXCLUDE (SUBJAREA , "MATH") OR EXCLUDE (SUBJAREA , "ENVI") OR EXCLUDE (SUBJAREA , "NEUR") OR EXCLUDE (SUBJAREA , "PHYS") OR EXCLUDE (SUBJAREA , "BIOC") OR EXCLUDE (SUBJAREA , "DECI") OR EXCLUDE (SUBJAREA , "BUSI") OR EXCLUDE (SUBJAREA , "AGRI") OR EXCLUDE (SUBJAREA , "MULT") OR EXCLUDE (SUBJAREA , "MATE") OR EXCLUDE (SUBJAREA , "ENER") OR EXCLUDE (SUBJAREA , "ECON") OR EXCLUDE (SUBJAREA , "PHAR") OR EXCLUDE (SUBJAREA , "DENT") OR EXCLUDE (SUBJAREA , "CHEM") OR EXCLUDE (SUBJAREA , "CENG")) AND (EXCLUDE (DOCTYPE , "ch") OR EXCLUDE (DOCTYPE , "bk") OR EXCLUDE (DOCTYPE , "cr") OR EXCLUDE (DOCTYPE , "cp")) AND (LIMIT-TO (LANGUAGE , "English")) AND (EXCLUDE (EXACTKEYWORD , "2-year-old Children") OR EXCLUDE (EXACTKEYWORD , "APPs") OR EXCLUDE (EXACTKEYWORD , "AR System") OR EXCLUDE (EXACTKEYWORD , "Actor-network Theory") OR EXCLUDE (EXACTKEYWORD , "Adaptation, Psychological") OR EXCLUDE (EXACTKEYWORD , "Addiction") OR EXCLUDE (EXACTKEYWORD , "Adolescents") OR EXCLUDE (EXACTKEYWORD , "Adult Mediation") OR EXCLUDE (EXACTKEYWORD , "Affordance") OR EXCLUDE (EXACTKEYWORD , "Anticipatory Guidance") OR EXCLUDE (EXACTKEYWORD , "Association") OR EXCLUDE (EXACTKEYWORD , "Association Experiment") OR EXCLUDE (EXACTKEYWORD , "Ation") OR EXCLUDE (EXACTKEYWORD , "Bi-factor Analysis") OR EXCLUDE (EXACTKEYWORD , "Bi/Multilingual Education") OR EXCLUDE (EXACTKEYWORD , "Bidirectionality") OR EXCLUDE (EXACTKEYWORD , "Blended Learning") OR EXCLUDE (EXACTKEYWORD , "Brain Injuries, Traumatic") OR EXCLUDE (EXACTKEYWORD , "COVID-19") OR EXCLUDE (EXACTKEYWORD , "CT (Computerized Tomography)") OR EXCLUDE (EXACTKEYWORD , "Ceaseless Parenting") OR EXCLUDE (EXACTKEYWORD , "Cerebrovascular Accident") OR EXCLUDE (EXACTKEYWORD , "Child & Adolescent Mental Health") OR EXCLUDE (EXACTKEYWORD , "Child Behavior") OR EXCLUDE (EXACTKEYWORD , "Child Protection") OR EXCLUDE (EXACTKEYWORD , "Child With ADHD") OR EXCLUDE (EXACTKEYWORD ,

		"Child-effect") OR EXCLUDE (EXACTKEYWORD , "Children With ADHD") OR EXCLUDE (EXACTKEYWORD , "Children With Acquired Brain Injury") OR EXCLUDE (EXACTKEYWORD , "Chronic Illness") OR EXCLUDE (EXACTKEYWORD , "Clase Colaborativa") OR EXCLUDE (EXACTKEYWORD , "Clinical Article") OR EXCLUDE (EXACTKEYWORD , "Collegial Learning") OR EXCLUDE (EXACTKEYWORD , "Collaborative Class") OR EXCLUDE (EXACTKEYWORD , "Collaborative") OR EXCLUDE (EXACTKEYWORD , "Cognitive Remediation Therapy") OR EXCLUDE (EXACTKEYWORD , "Cognitive Remediation") OR EXCLUDE (EXACTKEYWORD , "Cognitive Load Theory") OR EXCLUDE (EXACTKEYWORD , "Cognition Assessment") OR EXCLUDE (EXACTKEYWORD , "Cloud Computing") OR EXCLUDE (EXACTKEYWORD , "Computer Addiction") OR EXCLUDE (EXACTKEYWORD , "Computer Assisted Therapy") OR EXCLUDE (EXACTKEYWORD , "Control") OR EXCLUDE (EXACTKEYWORD , "Cross-sectional Study") OR EXCLUDE (EXACTKEYWORD , "Culture") OR EXCLUDE (EXACTKEYWORD , "Cultural Learning") OR EXCLUDE (EXACTKEYWORD , "Damage") OR EXCLUDE (EXACTKEYWORD , "Desktop Computer-hybrid Computers") OR EXCLUDE (EXACTKEYWORD , "Human") OR EXCLUDE (EXACTKEYWORD , "Humans") OR EXCLUDE (EXACTKEYWORD , "Male") OR EXCLUDE (EXACTKEYWORD , "Female") OR EXCLUDE (EXACTKEYWORD , "Mass Medium") OR EXCLUDE (EXACTKEYWORD , "Adult") OR EXCLUDE (EXACTKEYWORD , "Virtual Reality") OR EXCLUDE (EXACTKEYWORD , "Personal Digital Assistant") OR EXCLUDE (EXACTKEYWORD , "Older Adults") OR EXCLUDE (EXACTKEYWORD , "Neurorehabilitation") OR EXCLUDE (EXACTKEYWORD , "Motivation") OR EXCLUDE (EXACTKEYWORD , "Information Dissemination") OR EXCLUDE (EXACTKEYWORD , "Computer Aided Instruction") OR EXCLUDE (EXACTKEYWORD , "Computers, Handheld") OR EXCLUDE (EXACTKEYWORD , "Cognitive Dysfunction") OR EXCLUDE (EXACTKEYWORD , "Cognitive Defect") OR EXCLUDE (EXACTKEYWORD , "Books") OR EXCLUDE (EXACTKEYWORD , "Book") OR EXCLUDE (EXACTKEYWORD , "Attention") OR EXCLUDE (EXACTKEYWORD , "Adolescent"))
	"tablet*" AND "child*" AND "collaboration"	(TITLE-ABS-KEY ("tablet*") AND TITLE-ABS-KEY ("child*") AND TITLE-ABS-KEY ("collaboration") AND NOT TITLE-ABS-KEY ("youth") OR TITLE-ABS-KEY ("adolescent") OR TITLE-ABS-KEY ("autism")) AND PUBYEAR > 2013 AND PUBYEAR < 2025 AND (EXCLUDE (SUBJAREA , "MEDI") OR EXCLUDE (SUBJAREA , "ENGI") OR EXCLUDE (SUBJAREA , "HEAL") OR EXCLUDE (SUBJAREA , "NURS") OR EXCLUDE (SUBJAREA , "MATH") OR EXCLUDE (SUBJAREA , "ENVI") OR EXCLUDE (SUBJAREA , "NEUR") OR EXCLUDE (SUBJAREA , "PHYS") OR EXCLUDE (SUBJAREA , "BIOC") OR EXCLUDE (SUBJAREA , "DECI") OR EXCLUDE (SUBJAREA , "BUSI") OR EXCLUDE (SUBJAREA , "AGRI") OR EXCLUDE (SUBJAREA , "MULT") OR EXCLUDE (

		SUBJAREA , "MATE") OR EXCLUDE (SUBJAREA , "ENER") OR EXCLUDE (SUBJAREA , "ECON") OR EXCLUDE (SUBJAREA , "PHAR") OR EXCLUDE (SUBJAREA , "DENT") OR EXCLUDE (SUBJAREA , "CHEM") OR EXCLUDE (SUBJAREA , "CENG")) AND (EXCLUDE (DOCTYPE , "ch") OR EXCLUDE (DOCTYPE , "bk") OR EXCLUDE (DOCTYPE , "cr") OR EXCLUDE (DOCTYPE , "cp")) AND (LIMIT-TO (LANGUAGE , "English")) AND (EXCLUDE (EXACTKEYWORD , "Ants") OR EXCLUDE (EXACTKEYWORD , "Associated Sets") OR EXCLUDE (EXACTKEYWORD , "Ation") OR EXCLUDE (EXACTKEYWORD , "Bidirectional Interaction") OR EXCLUDE (EXACTKEYWORD , "Child With ADHD") OR EXCLUDE (EXACTKEYWORD , "Childhood Art") OR EXCLUDE (EXACTKEYWORD , "Children With ADHD") OR EXCLUDE (EXACTKEYWORD , "Children With Developmental Delays") OR EXCLUDE (EXACTKEYWORD , "Chinese Language Learning") OR EXCLUDE (EXACTKEYWORD , "Computer Science") OR EXCLUDE (EXACTKEYWORD , "Complex Systems") OR EXCLUDE (EXACTKEYWORD , "Developmental Disorders") OR EXCLUDE (EXACTKEYWORD , "Developmental Delay") OR EXCLUDE (EXACTKEYWORD , "Information Access") OR EXCLUDE (EXACTKEYWORD , "Information Science") OR EXCLUDE (EXACTKEYWORD , "Information Systems") OR EXCLUDE (EXACTKEYWORD , "Information Visualization") OR EXCLUDE (EXACTKEYWORD , "Language Disorders") OR EXCLUDE (EXACTKEYWORD , "Land Use Planning") OR EXCLUDE (EXACTKEYWORD , "Land Use") OR EXCLUDE (EXACTKEYWORD , "Librarianship/library Management") OR EXCLUDE (EXACTKEYWORD , "Library And Information Science") OR EXCLUDE (EXACTKEYWORD , "Library And Information Services") OR EXCLUDE (EXACTKEYWORD , "M-learning") OR EXCLUDE (EXACTKEYWORD , "Population Geography") OR EXCLUDE (EXACTKEYWORD , "Positive Interdependence") OR EXCLUDE (EXACTKEYWORD , "Program Diagnostics") OR EXCLUDE (EXACTKEYWORD , "Radio Frequency Identification (RFID)") OR EXCLUDE (EXACTKEYWORD , "RFID") OR EXCLUDE (EXACTKEYWORD , "S") OR EXCLUDE (EXACTKEYWORD , "Scientific Literacy") OR EXCLUDE (EXACTKEYWORD , "Separation"))
	"ipad*" AND "child*" AND "social"	(TITLE-ABS-KEY ("ipad*") AND TITLE-ABS-KEY ("child*") AND TITLE-ABS-KEY ("social") AND NOT TITLE-ABS-KEY ("youth") OR TITLE-ABS-KEY ("adolescent") OR TITLE-ABS-KEY ("autism")) AND PUBYEAR > 2013 AND PUBYEAR < 2025 AND (EXCLUDE (SUBJAREA , "MEDI") OR EXCLUDE (SUBJAREA , "ENGI") OR EXCLUDE (SUBJAREA , "HEAL") OR EXCLUDE (SUBJAREA , "NURS") OR EXCLUDE (SUBJAREA , "MATH") OR EXCLUDE (SUBJAREA , "ENVI") OR EXCLUDE (SUBJAREA , "NEUR") OR EXCLUDE (SUBJAREA , "PHYS") OR EXCLUDE (SUBJAREA , "BIOC") OR EXCLUDE (SUBJAREA , "DECI") OR EXCLUDE (SUBJAREA , "BUSI") OR EXCLUDE (SUBJAREA , "AGRI") OR

		EXCLUDE (SUBJAREA , "MULT") OR EXCLUDE (SUBJAREA , "MATE") OR EXCLUDE (SUBJAREA , "ENER") OR EXCLUDE (SUBJAREA , "ECON") OR EXCLUDE (SUBJAREA , "PHAR") OR EXCLUDE (SUBJAREA , "DENT") OR EXCLUDE (SUBJAREA , "CHEM") OR EXCLUDE (SUBJAREA , "CENG")) AND (EXCLUDE (DOCTYPE , "ch") OR EXCLUDE (DOCTYPE , "bk") OR EXCLUDE (DOCTYPE , "cr") OR EXCLUDE (DOCTYPE , "cp")) AND (LIMIT-TO (LANGUAGE , "English")) AND (EXCLUDE (EXACTKEYWORD , "Actor--network Theory") OR EXCLUDE (EXACTKEYWORD , "Activity Schedules") OR EXCLUDE (EXACTKEYWORD , "Activity Photos") OR EXCLUDE (EXACTKEYWORD , "Active Support") OR EXCLUDE (EXACTKEYWORD , "Accuracy") OR EXCLUDE (EXACTKEYWORD , "COVID-19") OR EXCLUDE (EXACTKEYWORD , "Child Labour") OR EXCLUDE (EXACTKEYWORD , "Cognition Disorders") OR EXCLUDE (EXACTKEYWORD , "Cognitive Defect") OR EXCLUDE (EXACTKEYWORD , "Male") OR EXCLUDE (EXACTKEYWORD , "Human") OR EXCLUDE (EXACTKEYWORD , "Female") OR EXCLUDE (EXACTKEYWORD , "Humans") OR EXCLUDE (EXACTKEYWORD , "Hand Held Computers") OR EXCLUDE (EXACTKEYWORD , "Reading Fluency") OR EXCLUDE (EXACTKEYWORD , "Rapid Eating") OR EXCLUDE (EXACTKEYWORD , "Normal Human") OR EXCLUDE (EXACTKEYWORD , "Neuropsychological Tests") OR EXCLUDE (EXACTKEYWORD , "Neuropsychological Test") OR EXCLUDE (EXACTKEYWORD , "Moral Judgment Development") OR EXCLUDE (EXACTKEYWORD , "Moral And Conventional Distinctions") OR EXCLUDE (EXACTKEYWORD , "Higher Education") OR EXCLUDE (EXACTKEYWORD , "Food Stuffing") OR EXCLUDE (EXACTKEYWORD , "Feedforward") OR EXCLUDE (EXACTKEYWORD , "English Languages") OR EXCLUDE (EXACTKEYWORD , "Elementary Education") OR EXCLUDE (EXACTKEYWORD , "Education, Special") OR EXCLUDE (EXACTKEYWORD , "Covid-19"))
	"ipad*" AND "child*" AND "communication"	(TITLE-ABS-KEY ("ipad*") AND TITLE-ABS-KEY ("child*") AND TITLE-ABS-KEY ("communication") AND NOT TITLE-ABS-KEY ("youth") OR TITLE-ABS-KEY ("adolescent") OR TITLE-ABS-KEY ("autism")) AND PUBYEAR > 2013 AND PUBYEAR < 2025 AND (EXCLUDE (SUBJAREA , "MEDI") OR EXCLUDE (SUBJAREA , "ENGI") OR EXCLUDE (SUBJAREA , "HEAL") OR EXCLUDE (SUBJAREA , "NURS") OR EXCLUDE (SUBJAREA , "MATH") OR EXCLUDE (SUBJAREA , "ENVI") OR EXCLUDE (SUBJAREA , "NEUR") OR EXCLUDE (SUBJAREA , "PHYS") OR EXCLUDE (SUBJAREA , "BIOC") OR EXCLUDE (SUBJAREA , "DECI") OR EXCLUDE (SUBJAREA , "BUSI") OR EXCLUDE (SUBJAREA , "AGRI") OR EXCLUDE (SUBJAREA , "MULT") OR EXCLUDE (SUBJAREA , "MATE") OR EXCLUDE (SUBJAREA , "ENER") OR EXCLUDE (SUBJAREA , "ECON") OR EXCLUDE (SUBJAREA , "PHAR") OR EXCLUDE (SUBJAREA , "DENT") OR EXCLUDE (SUBJAREA , "CHEM") OR EXCLUDE (SUBJAREA , "CENG")) AND

		(EXCLUDE (DOCTYPE , "ch") OR EXCLUDE (DOCTYPE , "bk") OR EXCLUDE (DOCTYPE , "cr") OR EXCLUDE (DOCTYPE , "cp")) AND (LIMIT-TO (LANGUAGE , "English")) AND (EXCLUDE (EXACTKEYWORD , "ASD") OR EXCLUDE (EXACTKEYWORD , "Virtual Tour") OR EXCLUDE (EXACTKEYWORD , "Tracing Effect") OR EXCLUDE (EXACTKEYWORD , "Struggling Readers") OR EXCLUDE (EXACTKEYWORD , "Special Needs") OR EXCLUDE (EXACTKEYWORD , "Higher Education") OR EXCLUDE (EXACTKEYWORD , "Engineering Education") OR EXCLUDE (EXACTKEYWORD , "Clause Construction") OR EXCLUDE (EXACTKEYWORD , "COVID-19"))
	"ipad*" AND "child*" AND "collaboration"	(TITLE-ABS-KEY ("ipad*") AND TITLE-ABS-KEY ("child*") AND TITLE-ABS-KEY ("collaboration") AND NOT TITLE-ABS-KEY ("youth") OR TITLE-ABS-KEY ("adolescent") OR TITLE-ABS-KEY ("autism")) AND PUBYEAR > 2013 AND PUBYEAR < 2025 AND (EXCLUDE (SUBJAREA , "MEDI") OR EXCLUDE (SUBJAREA , "ENGI") OR EXCLUDE (SUBJAREA , "HEAL") OR EXCLUDE (SUBJAREA , "NURS") OR EXCLUDE (SUBJAREA , "MATH") OR EXCLUDE (SUBJAREA , "ENVI") OR EXCLUDE (SUBJAREA , "NEUR") OR EXCLUDE (SUBJAREA , "PHYS") OR EXCLUDE (SUBJAREA , "BIOC") OR EXCLUDE (SUBJAREA , "DECI") OR EXCLUDE (SUBJAREA , "BUSI") OR EXCLUDE (SUBJAREA , "AGRI") OR EXCLUDE (SUBJAREA , "MULT") OR EXCLUDE (SUBJAREA , "MATE") OR EXCLUDE (SUBJAREA , "ENER") OR EXCLUDE (SUBJAREA , "ECON") OR EXCLUDE (SUBJAREA , "PHAR") OR EXCLUDE (SUBJAREA , "DENT") OR EXCLUDE (SUBJAREA , "CHEM") OR EXCLUDE (SUBJAREA , "CENG")) AND (EXCLUDE (DOCTYPE , "ch") OR EXCLUDE (DOCTYPE , "bk") OR EXCLUDE (DOCTYPE , "cr") OR EXCLUDE (DOCTYPE , "cp")) AND (LIMIT-TO (LANGUAGE , "English")) AND (EXCLUDE (EXACTKEYWORD , "ASD") OR EXCLUDE (EXACTKEYWORD , "Virtual Tour") OR EXCLUDE (EXACTKEYWORD , "Tracing Effect") OR EXCLUDE (EXACTKEYWORD , "Struggling Readers") OR EXCLUDE (EXACTKEYWORD , "Special Needs") OR EXCLUDE (EXACTKEYWORD , "Higher Education") OR EXCLUDE (EXACTKEYWORD , "Engineering Education") OR EXCLUDE (EXACTKEYWORD , "Clause Construction") OR EXCLUDE (EXACTKEYWORD , "COVID-19"))
