# Supplementary material for: The impact of touchscreen digital exposure on children’s social development and communication: a systematic review
Source: Front Psychol. 2025 Oct 13;16:1613625. doi: 10.3389/fpsyg.2025.1613625 (PMC12557575; doi:10.3389/fpsyg.2025.1613625)
Supplement: Supplementary file 2 [file Data_Sheet_2.pdf]

Appendix B: Characteristics of reviewed studies

A/A	Article / Authors	Year	Country	Sample size/ no of participants	Setting (education, family, museum, other)	Study design	Key findings		Touchscreen device								
								Television	Portable computer	Mobile phone/smartphone	Tablets	Interactive White Board (IWB)	Interactive tabletop	Mutli-touch tables	Video game console	ipod	Museum exhibits
1.	Exploring modalities and functions of social interaction initiated by toddlers in the U.S. during touchscreen play. *Journal of Children and Media* / Isabelle Gross & Su-hua Wang	2024	USA	29 children parents dyads	University laboratory	Children: played a non educational game on a touchscreen<br><br>Parents: questionnaires and surveys	The results indicated modality differences. The toddlers tended to initiate verbal interactions in the middle segment of gaming and nonverbal interactions at the beginning.				X						
2.	Touchscreen apps for child creativity: An 980 evaluation of creativity apps designed for young children. *Computers & Education*, 981 201*(*104811) / Booton, S. A, Kolancali, P., and Murphy, V. A.	2023	UK	152 (android apps aiming at young children)	Online	Correlational design was used	Quality ratings for creativity were low for both age groups.				X						
3.	Vietnamese pre-schoolers' tablet use and early childhood learning: an ecological investigation. *Journal of Children and Media,* 13(3), pp. 241-259 / Becky Pham , Sun Sun Lim	2019	Singapore	42 mother-child dyads	Home	Semi-structured, face-to-face interviews, and observations with note-taking and photo-taking	Vietnamese parents play a central role in their preschoolers' tablet use for learning. Due to their dependence on their caregivers, the preschoolers' access to tablets and content were initiated and heavily influenced by their parents' supervision.<br><br>Vietnamese parents' monitoring of their preschoolers' touchscreen device use is contextualised within the unique media landscape of Vietnam, where zealous adoption of foreign digital technologies is undermined by a dearth of local social scaffolding.				X						
4.	Emotion Matters: What Happens Between Young Children and Parents in a Touchscreen World. *International Journal of Communication,* 11, 561–580 / Hogeun Seo Claire Shinhea Lee	2017	USA	10 children<br><br>20 parents	Coffee shops, restaurants, home	Ethnographic interviews	Parents engaged in restrictive and technical mediation, though they often failed to effectively manage their children's media use due to practical challenges.			X	X						
5.	Contingent experience with touchscreens promotes<br><br>parent-child conversations. *Cognitive*	2021	Japan	36 children and their parents	Laboratory	Parents: survey<br><br>Children: app	Both mothers and children talked more often when the touchscreen provided con- tingency for their touches. Furthermore, the numbers of maternal and child utterances were related when playing with contingent touches but not with				X						

	development, 21 / Yuko Okumura, Tessei Kobayashi						non-contingent touches or when viewing the screen without touching it.										
6.	Evaluating Simultaneous Visual Instructions with Kindergarten Children on Touchscreen Devices. International Journal of Human–Computer Interaction, 36:1, 41-54 / Vicente Nacher, Fernando Garcia-Sanjuan & Javier Jaen	2020	Spain	75 children	School	Experiment/ observation	Kindergarten children effectively understood and interacted with simultaneous visual prompts in a game-based application, successfully completing tasks and gesture sequences without major issues. The prompts also reduced technical confusion and fostered learning-focused dialogue, with no observed gender differences in engagement or effectiveness.				X						
7.	Multimodal participation frameworks during young children's collaborative drawing on paper and on the iPad, *Thinking Skills and Creativity, 29,* 1-11 / Mona Sakr	2018	UK	12 children	School	Multimodal frameworks<br><br>Video observations	The study identified distinct patterns of multimodal interaction during collaborative drawing on paper and iPads, shaped by the affordances of each medium. While the iPad's dynamic visuals appeared to sustain attention and encourage collaborative engagement, traditional pens sometimes hindered fluid turn-taking and disrupted the collaborative flow.				X						
8.	Examination of the relationship between technology use of 5–6 year-old children and their social skills and social status. *Early Child Development and Care*, 188(2), pp. 168–182/ Hülya Gülay Ogelman, Hande Güngör, Özlem Körükçu and Hatice Erten Sarkaya	2016	Turkey	162 children and their parents	School and home	Relational descriptive study<br><br>Correlational survey	The study found no predictive relationship between children's use of technological devices and their social skills, offering novel insight for the design of guidance services. It emphasizes the need for adult-supervised, developmentally appropriate use of technology in early childhood. Such regulation should ensure that digital engagement supports rather than hinders children's overall development and well-being.	X	X	X							
9.	Smartphones distract parents from cultivating feelings of connection when spending time with their children. *Journal of Social and Personal Relationships.* 36, pp. 1619-1639 / Kostadin Kushlev & Elizabeth W. Dunn	2018	USA	200 parents (study 1)<br><br>292 parents (study 2)	Science museum	Field experiment	Study 1 offers preliminary experimental evidence that frequent smartphone uses by parents during time spent with their children is linked to reduced feelings of attentiveness and social connectedness. The findings also highlight the absence of a consistent "neutral" level of phone use, suggesting that device engagement varies significantly with context, thereby challenging the usefulness of no-instruction control conditions in related research.<br><br>Study 2 demonstrates that phone use can diminish the benefits of time spent with children by			X	X						

							reducing the quality of parental attention. When parents reported lower subjective attention due to phone use, they also experienced a decreased sense of social connection during those interactions.										
10.	The association between parent-child technology interference and cognitive and social-emotional development in preschool-aged children. *Child: Care, Health and Development,* 47(4), pp /477-483 / Valerie Carson & Nicholas Kuzik	2021	Canada	100 children and their parents	Sport camps & school & home	Survey & apps tasks	Frequent parent–child interruptions caused by smartphone use are linked to lower response inhibition and emotional self-regulation in children, along with increased externalizing and internalizing behaviors. However, the observed effect sizes were small.	X	X	X					X	X	
11.	Why Do Iranian Preschool-Aged Children Spend too Much Time in Front of Screens? A Preliminary Qualitative Study. *Children (Basel),* 10(1193) / Bita Shalani, Parviz Azadfallah, Hojjatollah Farahani & Serge Brand	2023	Iran	20 parents	School	Qualitative study interviews	Screen time (ST) is a multidimensional behavior influenced by a range of factors, including individual and child-specific characteristics, as well as familial, societal, and cultural influences. Its emergence and persistence are shaped by the interplay of multiple contributing elements.	X		X	X						
12.	The relationship between video games and social–emotional delay in Chinese rural preschoolers: A comparison of five types of media. *Child, Care, Health andDevelopment,* 50(5) / He Gou & Yinglong Yang	2024	China	1182 parents	Six provinces (local community)	Online survey<br><br>Parent report screening tool on children’s social-emotional developmental delay	Children's use of television, computers, tablets, or smartphones was not significantly associated with social–emotional developmental delays. However, increased time spent on game consoles was linked to a higher risk of social–emotional delay.									X	
13.	Watching them grow: Intergenerational video calling among transnational families in the age of smartphones. *Global Networks*. 22, pp. 119-133 / Ipek Demirsu	2022	Italy	30 mothers	School	Online semi-structured interviews	Research has demonstrated that routine digitalised family practices establish a circumambient‘setting’ in which children, even when not actively participating in conversations, remain involved in the family socialization process and learn through observing parent–grandparent interactions.			X							
14.	Screen-play: An observational study of the effect of screen media on Children's play in a museum setting.<br><br>Computers in Human Behavior, Volume 132 / Jane E. Shawcroft,	2022	USA	150 children	Museum	Micro-longitudinal<br><br>Observation	Observations indicate that children in museum settings rarely engaged with personal screen media, and that digital screens integrated into exhibits had minimal influence on their social and emotional play expression.			X	X						X

	Megan Gale, Katey Workman, Virginia Leiter, McKell Jorgensen-Wells, and Alexander C. Jensen																
15.	Parental Perception of the Social and Physical Environment Contributes to Gender Inequalities in Children's Screen Time. *Journal of Physical Activity and Health*, 19(2), pp. 108-117 /Daniela Rodrigues, Helena Nogueira, Augusta Gama, Aristides M. Machado-Rodrigues, Maria-Raquel G. Silva, Vítor Rosado-Marques, and Cristina Padez	2022	Portugal	6347 children<br><br>parents	Three regions	Survey to parents (questionnaire)	Neighbourhood features were more correlated with girls' screen time than boys', particularly among younger children. Also, more social than physical characteristics of the neighborhood were positively associated with children's use of television and mobile devices.	X	X	X	X			X	X		
16.	Smart device usage in early childhood is differentially associated with fine motor and language development. *Acta Paediatrica,*108(5), pp. 903-910 / Jin-Hwa Moon, Sang Yeon Cho, Sung Min, Lim Joo Hyung Roh, Min Sook Koh, Yong Joo Kim & Eunwoo Nam	2019	Korea	117 children & parents	School	Parental online survey (questionnaire) and Korean-developmental screening test<br><br>Children: Preschool receptive-expressive language scale diagnostic tool	Factors related to smart device use were found to be associated with early childhood and language development, varying across age groups and developmental domains such as fine motor, language, and social skills. Among three-year-olds, increased screen time was linked to lower language development, while more frequent use correlated with stronger fine motor skills; appropriate usage was also associated with enhanced social competence.	X		X	X						
17.	Using tablets in free play: The implementation of the digital play framework in Greece. *British Journal of Educational Technology*, 49, pp. 928-942 / Maria Hatzigianni , Athanasios Gregoriadis, Ioanna Karagiorgou & Sofia Chatzigeorgiadou	2018	Greece	16 children	School	Semi-structured observation scale	Findings from the study offer useful insights into how children learn to use digital devices.				X						
18.	The association between smartphone and tablet usage and children development. *Acta Psychologica*. 228:103646 / Supattra Chaibal & Salinee Chaiyakul	2022	Thailand	85 caregivers	Home	Interviews	It found a significant link between screen time and gross motor development ($p = 0.036$), while other developmental areas showed no such correlation. Children average about 83 minutes of smartphone use daily. Family income influenced some outcomes, though most caregivers reported no relevant medical history. The findings suggest screen time may affect certain developmental aspects, warrant further research.			X	X						
19.	Doll play prompts social thinking and social talking: Representations of internal state language	2022	UK	33 children	A laboratory or	Voice recording analysed using generalized estimating	The study found that children used internal state language (ISL) more frequently in joint play conditions compared to solo play conditions,				X			X	X		

	in the brain. *Developmental Science.* 25: e13163 / Salim Hashmi Ross E. Vanderwer,Amy L. Paine & Sarah A. Gerson				experimental setting	equations (GEEs).	indicating that social context influences language production. Additionally, children were more talkative during doll play than tablet play, suggesting that the type of play also affects communication.										
20.	Types and contexts of child mobile screen use and associations with early childhood behavior. *Early Childhood Research Quarterly*, 70, pp. 274-286 / Sumudu R. Mallawaarachchi, Jeromy Anglim, Sharon Horwood	2024	Australia	536 parents	Preschool<br><br>childcare	A parent-reported survey to estimate children's mobile screen use and its social contexts.	The study found that toddlers spent about 33 minutes daily on mobile screens, mostly watching programs (56%). Surprisingly, less program viewing was linked to more externalizing behavior after one year. High dropout rates limit conclusions about long-term effects.			X							
21.	The child-effect in the new media environment: challenges and opportunities for communication research, Journal of Children and Media, 10:1, 30-38 / Jan Van den Bulck, Kathleen Custers & Sara Nelissen	2016	Belgium	Papents-children<br><br>(no is not applicable)	Home	Theoretical overview	How children can influence their parents' media consumption through various forms of mediation, including restrictive, instructive, and social co-viewing. This dynamic underscore the need to explore how children's preferences and behaviors shape family media use, reversing traditional perspectives that focus solely on parental guidance.			X	X						
22.	They can interact, but can they learn? Toddlers' transfer learning from touchscreens and television. *Journal of Experimental Child Psychology*, 137, pp. 137-55. /Alecia Moser, Laura Zimmermann, Kelly Dickerson, Amanda Grenell, Rachel Barr, Peter Gerhardstein	2015	USA	172 children	Home	Experiment<br><br>Test	Children's action fidelity and goal imitation improved with age, with 3-year-olds demonstrating significantly higher performance than 2.5-year-olds. Additionally, transfer deficits were observed when testing conditions changed from 3D to 2D, affecting both age groups.	X			X						
23.	Under threes' play with tablets. *Journal of Early Childhood Research*, 19(3), pp. 283-297 /Jackie Marsh Jamal Lahmar, Lydia Plowman, Dylan Yamada-Rice, Julia Bishop Fiona Scot	2020	UK	945 parents	Home	Survey online<br><br>4 case studies of children interview	The survey found that 25% of under-threes in tablet-using households owned their own device, with slightly more boys (27%) than girls (23%) owning one. Additionally, middle and upper-class families were more likely to own iPads compared to working-class families, who tended to own cheaper tablets.				X						
24.	The most effective element in conceptualization is social interaction, not source or modality: A new model of the conceptual development in children. *Learning,*	2020	Turkey	90 children	School	Test<br><br>experiment<br><br>Talking-in-SCM condition	The study found that five-year-olds benefited significantly more from the support of SCM processes compared to seven-year-olds, indicating that younger children can conceptualize at a higher cognitive level with appropriate support. Significant differences				X						

	Culture and Social Interaction, 24 / Tolga Yıldız						were observed in performance between different experimental conditions, particularly favoring the Talking-in-SCM condition.										
25.	Differences in Performance on Developmental Tasks in Young Children Across Digital and Paper-Based Modalities: A Feasibility Trial. Journal of Developmental and Behavioural Pediatrics 39(9):726-735 /Allison G. Dempsey, Amy K. Barton, Andrea F. Duncan	2018	USA	80 parents	Pediatric primary care clinics	Survey	The study found no significant differences in children's performance across various developmental domains when tasks were presented in digital versus paper formats, and weekly access to technology was not associated with raw scores on any developmental tasks.			X	X						
26.	Categorization Activities in Norwegian Preschools: Digital Tools in Identifying, Articulating, and Assessing. *Frontiers in Psychology,* 10*(*973) /Pål Aarsan	2019	Norway	45 children<br><br>8 educators	Preschool	Video recordings	Digital literacy activities in early childhood education vary significantly based on social norms, digital tools, and participant interactions, highlighting the importance of both identifying and articulating concepts in educational settings.			X	X	X					
27.	Surveying preschool teachers' use of digital tablets: general and technology education related findings. *International Journal of Technology and Design Education,* 29, pp. 717–737 /Anna Otterborn, Konrad Schönborn, Magnus Hultén	2019	Sweden	327 educators	Preschool	Online survey activities	Preschool teachers recognize both educational benefits, such as participatory and collaborative learning, and disadvantages related to digital tablets, while also emphasizing the need for clearer curriculum guidelines and better management involvement in tablet implementation.			X	X						
28.	A brief tablet-based intervention benefits linguistic and communicative abilities in toddlers and preschoolers. n*pj Science of Learning.* 9(38) / Marcela Peña, Constanza Vásquez-Venegas, Patricia Cortés, Enrica Pittaluga, Mitzy Herrera, Esteban J. Pino, Raul G. Escobar, Ghislaine Dehaene-Lambertz & Pamela Guevara	2024	Chile	253 children	Four public preschool educational centers	Intervention<br><br>Evaluated children's responses to a touchscreen task involving word and letter-sound associations, scoring their accuracy and providing feedback based on their performance.	Toddlers and preschoolers showed higher accuracy in tasks during the encoding phase compared to the recognition phase, with significant improvements in performance.				X						
29.	Tablet for two: How do children collaborate around single player tablet games?. *International Journal of Human-Computer*	2021	UK	20 children	School	Video recordings	Collaboration among children using tablets is influenced by the design of the game, the nature of the task, and the dynamics of their interactions, with variations observed in collaboration				X						

	Studies, 145(102539) / Rowanne Fleck, Asimina Vasalou, Konstantina Stasinou					Intervention study	mechanisms between different child pairs.										
30.	Collaborating around digital table-tops: Children’s physical strategies from India, the UK and Finland. *Computer-Human Interaction,* 24(3) / Izdihar Jamil, Calkin Suero Montero, Mark Perry, Kenton O’Hara, Abhijit Karnik, Kaisa Pihlainen, Mark T. Marshall, Swathi Jha and Sanjay Gupta, Sriram Subramanian	2017	UK	247 children	Schools	Video observations	Children in India exhibited dynamic spatial positioning and frequent physical contact during collaboration, while those in the United Kingdom preferred fixed positioning with minimal contact, and Finnish children displayed a mix of both behaviors.				X						
31.	Building computer supported collaborative learning environments in early childhood classrooms. *Educational Technology Research and Development,* 68, pp. 249–267 /Donna Karno, Beth Hatcher	2020	USA	18 children	School (classroom setting)	Video observations	The Engage-2 multi-touch table facilitated high levels of social play and collaborative learning among young children, promoting problem-solving skills and collective agency while highlighting the importance of app design and group dynamics in their interactions.							X			
32.	RFID interactive tabletop application with tangible objects: exploratory study to observe young children’ behaviors*. Personal and Ubiquitous Computing.* 19, pp. 1259–1274 / Sebastien Kubicki, Marion Wolff Sophie Lepreux Christophe Kolski	2015	France	16 children	School	Observations and questionnaire	An interactive tabletop significantly enhances children's interactions and collaborative work, with positive perceptions from teachers across different age groups, despite some confusion among younger children regarding object colors.						X				
33.	Turn-taking and sense making: Children and professionals co-producing and revisiting multimodal books during transition from day-care to school. *Learning, Culture and Social Interaction.* 24(100385) /Ane Bjerre Odgaard	2020	Denmark	8 children<br><br>2 educators	School	Video observation	The activities surrounding multimodal books during transitions reveal a complex interplay between children's communicative agency and accountability, highlighting how turn-taking structures and sense-making processes are shaped by individual contributions and social interactions within educational settings.					X					
34.	Impact of different interaction protocols on group communication, satisfaction and learning outcomes of primary school children when	2020	Spain	180 children	School	Survey	There were no statistically significant differences in learning outcomes or satisfaction between the turn-taking and consensus protocols, although both treatments						X				

	using multitouch tabletops. *Computers & Education,* 152(103875) / David Roldan-Alvarez, Adrian Bacelo, Estefanía Martín, Pablo A. Haya						resulted in improved post-test scores compared to pre-tests.										
35.	Differences in Parent-Toddler Interactions with Electronic versus Print Books. *Pediatrics.* 143(4): e20182012 / Tiffany G. Munzer, Alison L. Miller, Heidi M. Weeks, Niko Kaciroti, Jenny Radesky	2019	USA	37 parents	University	Experimental,laboratory-based study<br><br>Video recording	Parents and toddlers engaged in more dialogic and book-related verbalizations, as well as higher collaborative book-reading scores, when using print books compared to enhanced or basic electronic books, indicating that print formats may facilitate richer interactions.				X	X					
36.	Investigating Young Children's Social Interactions During Digital Play. *Early Child Educational Journal* 50, pp. 1449-1459 / Leigh Disney, Gretchen Geng	2022	Australia	80 children	Childcare centres	Video observations	While most areas of children's engagement during digital play were rated above medium, social interaction through verbal communication was notably weak, raising concerns about language development amidst the influence of peers on children's self-confidence and competence in digital play.				X						
37.	Associations among child temperament, parenting, and young children's moral and conventional understanding: The moderating role of self-regulation. *Social Development*, 31, pp. 619–638 / Ha Na Yoo, Judith G. Smetan	2022	USA	57 child-parent dyads	From different settings	Survey<br><br>Semi-structured interviews	Child age and self-regulation, particularly as assessed by the Children's Behavior Questionnaire, significantly influence moral and social-conventional distinctions, while parental hostility and positive affect did not show significant effects. Interactions between child age and self-regulation, as well as negative affect, approached significance, suggesting developmental considerations in children's moral judgments.				X						
38.	Connected learning with media tools in kindergarten: an illustrative case. *Educational Media International*, 56*(*3), pp. 233-249 / Henriikka Vartiainen, Teemu Leinonen & Saara Nissine	2019	Finland	42 children<br><br>2 educators<br><br>6 childcare nurses and assistants	School	Interviews<br><br>Project portfolios<br><br>Observations	The shared objects of inquiry in the kindergarten were grounded in children's real-world experiences and collaborative problem-solving, facilitated by media tools that expanded the community of inquiry and led to the creation of meaningful artifacts.				X						
39.	Using iPad tablets for self-modeling with preschoolers: Videos versus photos. *Psychology in the Schools.* 54, pp. 821-836 / Dacia M. McCoy, Julie Q. Morrison, Dave W. Barnett, Hilary D.	2017	USA	3 children<br><br>3 educators	School	Intervention case<br><br>Video recordings	The intervention phases resulted in increased engagement and reduced off-task behaviors among the children, with variability in engagement decreasing compared to baseline, although some limitations were noted due to child absences and differences in intervention phase lengths.				X						

	Kalra, Lauren K. Donovan																
40.	Young Children Drawing Together on the iPad Versus Paper: How Collaborative Creativity is Shaped by Different Semiotic Resources. *International Journal of Education & the Arts,* pp.*1-26.* Mona Sakr	2019	UK	12 children	School (separated classroom)	Thematic analysis	The analysis suggests that drawing on the iPad can be more responsive and less subject to personal planning than drawing on paper. This difference is shaped by physical properties such as the touch-screen interface, but also emerges as a result of the cultural investment in drawing on paper as a form of 'self-expression', a notion that works to limit exploratory and collaborative engagement with the resources. Since participants were noticeably open to exploring new ideas together while drawing on the iPad, the researchers argue that we need to reassess the potentials of touch-screen tablets to support tasks of collaborative creativity in educational contexts.				X						
41.	How pedagogical relations in early years settings are reconfigured by interactive touchscreens. *British Journal of Educational Technology,* 53, pp. 58-76 / Robin Samuelsson, SaraPrice, Carey Jewitt	2021	Sweden	9 children<br><br>2 educators<br><br>1 assistant	School	Video<br><br>Photos<br><br>Field notes	While children's verbal communication decreased during iPad sessions compared to shared book readings, their bodily actions increased, indicating a shift in interaction dynamics and suggesting both opportunities for autonomy and potential drawbacks for linguistic development in early childhood education.				X						
42.	Impact of Shared iPads on Kindergarten Students' Collaboration and Engagement in Visual Storytelling Activities. *Early Childhood Education Journal,* 48, pp. 521–53 / Fatimah A. Dashti, Kawthar M. Habeeb	2020	State of Kuwait	40 children	School	Observations	Children's collaboration during activities on a shared iPad or paper was primarily influenced by personality rather than the method of drawing, with no significant differences in their interactions or preferences for using technology versus traditional materials.				X						
43.	Mathematical strategies and emergence of socially mediated metacognition within a multi-touch Dynamic Geometry Environment. *Educational Studies in Mathematics,* 112, pp. 289–307 /Stephen J. Hegedus, Yenny Otálora	2023	USA	6 children	School	Observations<br><br>Interviews	Learners can effectively access core mathematical concepts like covariation through collaborative strategies and socially mediated metacognition facilitated by multimodal environments.				X						
44.	Young pupils', their teacher's and classroom assistants' experiences of iPads in a Northern Ireland school: "Four and five years old, who would have thought they could do that?". *British*	2016	UK	27 children	School	Interviews	The pupils expressed positive experiences with iPads in enhancing their literacy and numeracy skills, fostering independent learning, motivation, and peer collaboration, although definitive improvements				X						

	Journal of Educational Technology, 47, pp. 1051-1064 / Linda Clarke and Lesley Abbott						could not be measured due to the lack of baseline data.										
45.	Developing numeracy skills using interactive technology in a play-based learning environment. *IJ STEM Ed* 5, 39 / Tess Miller	2018	Canada	13 children<br><br>1 educator	School	Experimental<br><br>Mixed methods<br><br>Conversations<br><br>Field notes<br><br>Experimental test	Small math gains and high engagement suggest interactive technology supports kindergarten math learning. App quality and appropriate difficulty were key—fun, creative apps boosted engagement, while overly hard ones led to disengagement.				X						
46.	Child–father creative text-making at home with crayons, iPad collage & PC. *Thinking Skills and Creativity*, 17, pp. 59-73 / N. Kucirkova, M. Sakr	2015	UK	1 child<br><br>1 parent	Home	Video observations	The digital and non-digital resources positively influenced the child's creativity and possibility of thinking, highlighting the father's supportive role in their collaborative creativity during text-making activities.		X		X						
47.	Digital funds of identity: understanding a young child's plurilingual development through mediagrams. *Innovation in Language Learning and Teaching,* 18(3), pp. 208-222 / Sabine Little & Kexin Cheng	2024	UK	1 child	Home	Interviews<br><br>Video recordings	Feifei, a three-year-old girl, demonstrates the ability to distinguish and use multiple languages—Mandarin, English, and Bahasa Indonesia—through her interactions with touchscreen activities, as evidenced by the analysis of family interviews, parent-recorded videos, and evolving mediagrams.				X						
48.	The Ghost in the Touchscreen: Social Scaffolds Promote Learning by Toddlers. *Child Development*, 88(6), pp. 2013-2025 / Laura Zimmermann, Herietta Lee, Rachel Barr, Alecia Moser, Peter Gerhardstein	2017	USA	52 children	University setting	Video recordings	Young children's learning is significantly influenced by social scaffolding, with older children performing better than younger ones, particularly in 3D ghost conditions compared to 2D, highlighting the importance of social contingency in media-based learning.	X									
49.	Becoming a high-fidelity – *super* – imitator: what are the contributions of social and individual learning? *Developmental Science*, 18, pp. 1025-1035 / Francys Subiaul, Eric M. Patterson, Brian Schilder, Elizabeth Renner and Rachel Barr	2015	USA	215 children	Museum	Observation	The effects of different learning conditions, specifically Trial-and-Error and Recall, on children's ability to learn and imitate sequences, revealing significant age-related differences in performance across various tasks.		X								

50.	Parent-child conversations about evolution in the context of an interactive museum display. *International Electronic Journal of Elementary Education*, 5(1), pp. 27–46 /Andrew Shtulman, Isabel Checa	2012	USA	49 parent-child dyads	Museum	Interview	The study found that higher parental engagement and the child's age positively influenced the accuracy of parent-child dyads' responses to evolutionary concepts, with parental engagement being a stronger predictor than child age.		X								
51.	Tangible digital storytelling and phygital reality:<br><br>benefits for inclusion and cooperation in young children Research on Education and Media, *15* (2), 41-50 /Angela Pasqualotto, Fabio Filosofi	2023	Switzerland	Not applicable	Combination of physical reality and digital reality in Tangible Digital Storytelling (TDST) activities	Storytelling remains one of the oldest teaching methods for children: the development of technology has given impetus to the creation of tangible digital storytelling, which combines programs with physical objects, creating a single field for learning.	Overall, the findings suggest that collaborative storytelling, particularly in the context of Tangible Digital Story Telling, offers significant benefits for students, including those with special needs.<br><br>This study considers the evolution of the storytelling practice, focusing specifically on tangible digital storytelling and its benefits in young children						X				
52.	Negotiating screen time: A mother's struggle over 'no screen time' with her infant son. *Journal of Early Childhood Literacy*, 20(3), pp. 524-550 / Laura Teichert	2020	USA	1 child<br><br>1 mother	Phenome nology & auto ethnography	Field notes	This study highlights the confusion and tension parents face in navigating screen time recommendations for young children amid conflicting guidance from pediatric organizations and media reports. The author, a mother, emphasizes that digital devices are integral to her family's daily life, used not just for communication but also for bonding and play. She advocates clearer definitions of "screen time" and "educational content," alongside more nuanced, family-informed guidelines that recognize the realities of digital life from infancy onward.	X	X								
53.	The impact of parents' smartphone use on language development in young children. *Child Development Perspectives*, 16, pp. 103–109 / Amanda J. Morris, Maria Laura Filippetti, Silvia Rigato	2022	UK	Parents and children<br><br>(not applicable)	Review of evidence of the effects of smartphone technoferenc e during parent–child interactions on language outcomes.	Exploration of technoference in existing research studies	Findings point toward negative outcomes in language development, but it is less clear what processes affect language outcomes. Gaze following, parental responsiveness, and joint attention are also reduced when parents use their smartphone, and all are critical to language development			X	X						
54.	Parents' perspectives: Children's use of technology in the Early Years. London: National Literacy Trust / Susie Formby	2014	UK	1028 parents	Online survey	Literacy Survey	Technology offers a route into reading for disadvantaged three to five-year-old children. Of children who have a touch screen at home, children of lower socioeconomic status are twice as likely to look at	X	X	X	X						

							stories daily (16.0% vs. 7.2%). We also found that poorer children who use both books and touch screens to look at stories are less likely to perform below the expected standard for their age than if they only look at books. Not only does technology offer a route into reading for disadvantaged children, we also found that children are more likely to enjoy reading more if they look at stories using both books and a touch screen, compared withusing books only (77.4% vs. 70.8%).										
55.	Assessing the educational potential and language content of touchscreen apps for preschool children. *Computers and Education Open,* 3:100102 / Gemma Taylor, Joanna Kolak, Sarah H. Norgate, Padraic Monaghan	2022	UK	Not applicable<br><br>apps evaluation with language content (N=76)	Virtual environment	Apps were coded using two complementary tools: (1) a questionnaire<br><br>for evaluating the educational potential of apps and (2) coding criteria for quantifying the app features, developed by Kolak et al. (2021)	The researchers argue that selecting children's apps based on the presence of a learning goal is a good first step for selecting an educational app for pre-school age children.			X	X						
56.	Selecting educational apps for preschool children: How useful are website app rating systems? *British Journal of Educational Technology*, 53, 1262–1282 / Gemma Taylor, Joanna Kolak, Padraic Monaghan, Eve M. Bent	2022	UK	Not applicable<br><br>apps evaluation	Virtual environment	Apps were coded using two complementary tools: (1) a questionnaire and (2) coding criteria	The findings of this study are of use to parents and early years' practitioners who can be made aware of how useful website rating systems are when compared to the research-based tools for assessing apps. The findings of this paper can increase their awareness of the strengths and weaknesses of the criteria that website rating systems use.				X						
57.	Children's Virtual Worlds and Friendships during the covid-19 Pandemic, Video. *Journal of Education and Pedagogy*, 5(1), pp. 1-18 / Gloria Quinones & Megan Adam	2021	Australia	2 children	Home (remotely)	Online platfroms	The study found that digital technology enabled children to engage in imaginative play and virtual games together, helping them share and negotiate roles across various platforms. To prevent social isolation during periods of social distancing, parents supported their children's use of these technologies. The digital tools allowed the children to sustain their friendship through emotional and visual connections in virtual environments. Overall, digital technology emerged as a valuable learning tool that fostered a collective social experience by				X						

							integrating real, imaginary, and virtual worlds.										
58.	Use of touchscreen technology by 0–3-year-old children: Parents' practices and perspectives in Norway, Portugal and Japan. *Journal of Early Childhood Literacy*, 20(3), pp. 551-573 / Maria Dardanou, Torstein Unstad, Rita Brito, Patricia Dias, Olga Fotakopoulou, Yoko Sakata, Jane O'Connor	2020	Norway	552 parents & grandparents	Online	Survey	Touchscreen technologies have become integrated into family life across all three countries studied, though notable differences and overlaps exist in parental attitudes toward young children's use. The findings reveal the emergence of new digital parenting behaviors, shaped by concerns around protection and control, and often expressed through screen-time restrictions. Parents' values and practices are influenced not only by dominant national discourses about childhood and technology but also by beliefs about the role of play and the educational potential of touchscreens in supporting early literacy and numeracy.			X							
59.	Closing the gap? Overcoming limitations in sociomaterial accounts of early literacy. *Journal of Early Childhood Literacy*, 20(1), pp. 111-133 / Cathy Burnett and Guy Merchant, Michelle M Neumann	2020	UK	Not applicable	School	Vignettes classroom practice	Social inequality is challenged by questioning the assumptions inherent in deficit models of children's literacy, while simultaneously advancing alternative approaches that highlight the generative potential of interactions among individuals and materials. This perspective emphasizes collaborative assemblages as a basis for reimagining literacy and promoting more equitable educational practices.			X	X						
60.	How self-generated labelling shapes transfer of learning during early childhood: The role of individual differences. *British Journal of Developmental Psychology*, 37(1), pp. 68-83 / Alecia Moser, Sarah Olsen, Sylvia N. Rusnak, Rachel Barr and Peter Gerhardstein	2019	USA	355 children	Virtual environment<br><br>laboratory-based, counterbalanced	A secondary data analysis of individual differences in self-generated labelling using data collected from a complex puzzle imitation task with	This analysis revealed that self-generated labelling was lower when the social demonstrator was removed and the task was presented on a touchscreen. This study is one of the first to examine self-generated labelling during a complex imitation task in toddlers and increases our understanding of the complexity of memory processing needed for imitation learning.	X									
61.	Toddlers on touchscreens: immediate effects of gaming and physical activity on cognitive flexibility of 2.5-year-olds in the US. *Journal of Children and Media*, 12(4), pp. 496–513 / Nick K. Antrilli & Su-hua Wang	2018	USA	78 children	Neighboring communities	Experiment<br><br>Questionnaire	Toddlers who participated in physical play outperformed their peers in a sorting task compared to those who either played a non-educational touchscreen game without social interaction or engaged in drawing for an equivalent duration. These findings indicate that, in the short term, physical activity may have a more positive impact on toddlers'				X						

							cognitive flexibility than solitary touchscreen play or drawing activities.										
62.	Early childhood educators’ attitudes and beliefs around the use of touchscreen technologies by children under three years of age. *British Journal of Educational Technology*, 49, pp. 883-895 / Maria Hatzigianni and Ioannis Kalaitzidi	2018	Australia	203 educators	University’s ECE centres	Online survey<br><br>Focgroups	The study found that while teachers were generally confident in their personal use of technology and open to innovation, they expressed uncertainty about the role of touchscreen technologies in supporting children's free play—a central principle in early childhood education. The findings underscore the importance of training and professional development, particularly when tailored to the everyday realities and interactions of educators working with very young children. Such targeted support is essential for helping teachers integrate technology meaningfully into early learning environments.		X	X							
63.	What’s next for research on young children’s interactive media? *Journal of Children and Media*, 10(1), pp. 54–62 / Georgene L. Troseth, Colleen E. Russo & Gabrielle A. Strouse	2016	Canada	Not applicable<br><br>Evaluation of research studies on interactivity	Each study reported was carried out in different settings	A selection of different methodologies were reported, based on all studies evaluated	The past decade has seen vast changes in children’s media, including the release of modern smartphones and tablets, as well as countless other digital devices specifically for children. The pace of new industry breakthroughs is likely to continue at an exponential rate and it is crucial that research stays current.	X	X	X	X						
64.	Learning Number with TouchCounts: The Role of Emotions and the Body in Mathematical Communication. *Tech Know Learn,* 19, pp. 81–99 / Nathalie Sinclair, Einat Heyd-Metzuyanim	2014	Canada	1 child<br><br>1 educator	School (separated classroom)	Interviews tasks with TouchCounts app	This study highlights how focusing on both cognitive and emotional dimensions of child–teacher–iPad interactions reveal the effectiveness of the TouchCounts app as a powerful learning tool. Its multi-touch capabilities allow children to engage in unique one-to-one-to-one correspondences between touch, counting, and object manipulation, expanding the conceptual understanding of numbers beyond traditional approaches. The app facilitates novel, embodied, and multimodal learning experiences that are not easily replicated in other environments, making it a meaningful and emotionally engaging resource for both teachers and learners.				X						

65.	Touchscreen generation: children's current media use, parental supervision methods and attitudes towards contemporary Media. A*cta Paediatrica,* 106(4), pp. 654-662 / Katarzyna Kostyrka-Allchorne, Nicholas R. Cooper, Andrew Simpson	2017	UK	90 parents	School & home	Survey (printed questionnaire)	The findings indicate that while traditional television remains the preferred media platform for young children, touchscreen devices like tablets are becoming increasingly popular and enable multi-screen use, posing new challenges for parental supervision. Parents themselves tend to use these newer media platforms less frequently for entertainment and are less familiar with digital game and app rating systems compared to film and television ratings. Nonetheless, their concerns about harmful media content—particularly violence and inappropriate language or behavior— align closely with those of researchers and clinicians.	X	X	X	X					X	
66.	One screen, many fingers: Young children's collaborative literacy play with digital puppetry apps and touchscreen technologies. Digital media and literacies special issue. Theory Into Practice, 54, 154–162 / Wohlwend, K.	2015	USA	2 children	Home	Audio-recording	There is a need to transform traditional reading and writing workshops into dynamic, media-rich "playshops" that support play-centered digital literacy. Early literacy pedagogies should encourage children to explore and create future literacies, rather than limiting learning to standardized test preparation. This involves fostering collaborative digital literacies and adopting teaching practices that promote playful, meaningful engagement with new technologies.		X		X						
67.	An effort to understand parents' media mediation roles and early childhood children's digital game addiction tendency: A descriptive correlational survey study. *Education Information Technology* 29, pp. 17825–17865 / Ceren Çalhan, İdris Göksu	2024	Turkey	433 parents	Online	Quantitative research<br><br>Relational screening model<br><br>Correlation survey	Parents' and children's screen time was negatively related to parental media mediation and positively linked to children's tendencies toward digital game addiction. Mothers were more likely than fathers to perceive their children as having signs of digital game addiction and were also more active in media mediation. Higher addiction tendencies were observed among male children, children with their own devices, those whose parents also play digital games, and those who primarily watch YouTube compared to children's television channels. Additionally, parents tended to impose more restrictions on daughters than on sons.	X	X	X	X			X	X		
68.	Screens' domestication in childhood: uses and parental mediation in city and rural contexts. *Comunicação e sociedade*. 37 / Carla Cruz, Catarina Franco,	2020	Portugal	20 children<br><br>8 parents	School	Focus groups and semi-structured interviews	Children from V.P.V. commonly use screens alone and show less awareness of parental rules, unlike Lisbon children who typically use screens with family members and report on clearer parental mediation. Across both groups, portable digital screens are mainly	X		X	X						

	Fábio Anunciação and Maria João Cunha					Comparative study of 2 areas (city-rural)	used at home during routine moments, with occasional use in public or social settings. Parents in both regions expressed feeling pressured to provide digital access to avoid excluding their children from their peer group's digital culture.										
69.	Technology and Media Use in Preschool Classrooms: Prevalence, Purposes, and Contexts. *Frontiers in Education,* 5*(*600305) / Rebecca A. Dore and Jaclyn M. Dynia	2020	USA	312 educators	Virtual environment	Online survey	This study suggests that technology is widely used in preschool classrooms, with teachers employing devices across varied contexts and for multiple educational purposes. Distinct patterns of technology use emerged, shaped by the frequency and purpose of use. These patterns were influenced by both teacher and program characteristics, highlighting implications for educational research and practice.	X	X	X	X						
70.	Tablet Computers in Early Science Education: Enriching Teacher–Child Interactions. *Early Childhood Education Journal /* Ueli Thomas Studhalter, Priska Jossen, Marco Seeli Annette Tettenborn	2024	Switzerland	2 educators<br><br>41 children	School	Mixed- methods approach videographic setup and science content-relevant interaction episodes	Integrating tablet computers into teacher–child interactions was found to extend the duration and depth of conversations, supporting sustained shared thinking and high-quality scaffolding. Teachers used open-ended prompts and subject-specific vocabulary to guide focused observations, while children demonstrated increased verbal participation. The study highlights how tablets can enhance interactive learning and counter concerns about digital isolation in early education settings.				X						
71.	"We Usually Go Out Instead, So That He Forgets About His Tablet" (Great-)Grandparental Mediation in the Generational Order. *International Journal of Communication***,** 16(8) / Carolina Martin	2022	Sweden	18 grandparents and great-grandparents	Home & community centers	Semi-structured interviews	The study highlights that grandparental mediation often centers on responsible media use and participatory learning, offering children valuable guidance in a less directive manner. Unlike parents, grandparents typically adopt a more indirect role shaped by intergenerational dynamics and respect for parental authority. Their approach aligns more closely with peer mediation, emphasizing co-use, mutual support, and shared experiences over control or instruction.				X						
72.	"I'm Just Playing iPad": Comparing Prekindergarteners' and Preservice Teachers' Social Interactions While Using Tablets for Learning', *Journal of Early Childhood*	2015	USA	18 children	School	Observations<br><br>Open-ended exploration of multiple center activities	Observations showed that both younger and older learners shifted from individual screen engagement to social interactions while using interactive technologies. Their verbal and physical responses reflected curiosity, emotional involvement, and a collaborative				X						

	Teacher Education, 36(4), pp. 362–378 / Holly Carrell Moore & Jennifer Keys Adair						effort to construct understanding and meaning during iPad app exploration.										
73.	Responding to Children’s Semiotic Repertoires in Collaborative Digital Storytelling. *Early Childhood Education Journal /* Shengjergji, Jenny Myrendal, Niklas Pramling	2024	Sweden	23 children<br>4 educators	School/ Digital storytelling activities	Video recordings	This study emphasizes the importance of working towards equity rather than equality. Hence, responsivity entails considering and responding to all semiotic repertoires through which children represent and communicate their continuous meaning-making.				X						
74.	Emerging Mobile Learning Pedagogy Practices: Using tablets and constructive apps in early childhood education. *Educational Media International*, 57(3), pp. 253-270 / Monika Tavernier & Xiao Hu	2020	Hong Kong	15 children<br>1 educator	School	Video & digital artifacts	This highlights cchildren's engagement and output quality in digital creation activities varied, with the most successful and motivating experiences linked to four key factors: connection to previous class activities, collaboration with preferred peers, use of favored tools, and opportunities to express personal interests. These factors reflect perceived competence, autonomy, and relatedness, suggesting that meaningful, personalized tasks enhance motivation and creative performance.				X						
75.	Using tablet computers in preschool: How does the design of applications influence participation, interaction and dialogues? *International Journal of Early Years Education /* Hanna Palmér	2015	Sweden	25 children<br>8 educators	School	Observations	This study suggests that to enhance children's participation and promote open dialogue in structured activities, digital applications with weak framing are preferable. While strong classification may help integrate mathematics into discussions, weak framing appears essential for fostering interaction and spontaneous dialogue between teachers and children—though such apps are rare and offer no guarantees of curriculum-aligned learning.				X						
76.	Turn-taking and sense making: Children and professionals co-producing and revisiting multimodal books during transition from day-care to school. *Learning, Culture and Social Interaction.* 24(100385) /Ane Bjerre Odgaar	2020	Denmark	8 children<br>2 educators	Daycare	Video observation	This study highlights how the social and dialogic foundations of individual learning paths are often overlooked, emphasizing the need for pedagogical efforts that foster community-building. It reveals a tension in tool-mediated digital activities between individualized, sequential task structures and richer, multi-voiced interactivities, offering insights into how digital technologies shape sense-making in early childhood education.				X	X					

77.	Broadening the Notion of Peer-To-Peer Interactions When Young Children Engage With Digital Technology. *Early Childhood Education Journal* / Andrea Nolan, Deborah Moore	2024	Australia	23 children<br><br>5 educators	School	Observation	This study broadens the understanding of peer-to-peer interactions among young children using digital technologies, highlighting that social engagement extends beyond overt cues like eye contact or vocalizations. It draws attention to subtle, often overlooked behaviors that signify interaction, urging adults to adopt a more nuanced perspective when observing children's digital play.				X						
78.	Print or iPad? Young Children's Text Type Shared Reading Preference and Behaviors in Comparison to Parent Predictions and At-home Practices. *Literacy Research and Instruction*, 59(4), pp. 324-345 / Lauren Eutsler & Julia Trotte	2020	USA	37 parents<br><br>37 children	University laboratory preschool	Social learning theory<br><br>Multiple case-study design<br><br>Parent survey<br><br>Observations (printed and digital book	Parent perceptions suggest a lack of awareness or knowledge of effective digital shared reading strategies to support their children's reading preferences. The findings underscore the importance of informing parents and teachers that both print and digital texts can successfully engage children during shared reading. While children showed slightly more attention to digital reading, they were generally engaged with the story in both formats.				X						
79.	Preschool Children and iPads: Observations of Social Interactions During Digital Play, Early Education and Development, 29:2, 207 – 228 / Sandra M. Lawrence	2018	USA	20 children	School	Observational field notes	Children predominantly engaged with closed-design apps, which supported practice-based play involving literacy, numeracy, and pattern recognition. However, the restrictive content limited opportunities for creative construction or collaborative pretend play.				X						
80.	Parent-child moments of meeting in art-making with collage, iPad, tuxpaint, and crayons. *International Journal of Education & the Arts*, 18(2) / Mona Sakr, Natalia Kucirkova	2017	UK	1 child<br><br>1 parent	Home	Observations	Different technologies play a distinct role in parent-child moments of meeting and outlines how these differences relate to the material resources that are used to make meaning and the social, agential and embodied experiences through which these meaning-making experiences unfold		X		X						
81.	Digital play: Exploring young children's perspectives on applications designed for preschoolers. *Global Studies of Childhood.* 6, pp. 388-399 / Irina Verenikina, Lisa Kervin, Maria Clara Rivera and Alison Lidbetter	2016	Australia	10 children<br><br>10 parents	Not reported	Play sessions (audio & visual recording)	The study highlights that social interaction is key to empowering children and enriching their digital play experiences. Young children responded positively to the apps, engaging in extended make-believe play while using both private and social speech. Personalization features significantly enhanced their engagement—particularly for boys.				X						

2024 =9
2023 =4
2022=11
2021=5

2020=15
2019=8
2018=8
2017=6
2016=5
2015=7
2014=2
