# Supplementary material for: The impact of touchscreen digital exposure on children’s social development and communication: a systematic review
Source: Front Psychol. 2025 Oct 13;16:1613625. doi: 10.3389/fpsyg.2025.1613625 (PMC12557575; doi:10.3389/fpsyg.2025.1613625)
Supplement: Supplementary file 3 [file Data_Sheet_3.pdf]

Appendix C: Sentiment analysis on the impact of the use of touchscreens on young children's social development

Positivity references (67)

Reference 1 - 0.25% Coverage

The present exploratory study is among the first to centre on the child's active role in social engagement as they navigate gaming.

Reference 2 - 0.26% Coverage

However, the article suggests that social interactivity through co-viewing may still play a significant role in supporting social skills.

Reference 3 - 0.19% Coverage

However, excessive screen time might also detract from opportunities for direct peer interactions.

Reference 4 - 0.49% Coverage

However, successful collaboration depends on teacher guidance and structured learning designs

Excessive and unsupervised use of technology can lead to passivity in social interactions and difficulties in peer relationships, potentially resulting in isolation.

Reference 5 - 0.23% Coverage

However, when used appropriately in educational settings, mobile technologies can enhance social and cognitive development.

Reference 6 - 0.23% Coverage

Higher technology interference was significantly associated with lower response inhibition and emotional self-regulation.

Reference 7 - 0.76% Coverage

The study highlights that children often prefer digital entertainment over social activities, leading to less face-to-face communication and cooperative experiences

Young children's risk of social–emotional delay was found to increase only when their time spent on game consoles increased, compared with time spent on other popular digital media such as television, computers, tablets, and smartphones.

Reference 8 - 0.53% Coverage

When the study results are examined, generally it is observed that use of mobile technology (television, portable computers, tablets, smart phones and all devices) on weekdays and at weekends did not significantly predict the frequency of children to realize their social skills.

Reference 9 - 0.37% Coverage

The fact that use of mobile technology had no effect upon the social skill level could be interpreted as the presence of different variables that may have a greater effect on the social skill level.

Reference 10 - 0.16% Coverage

However, excessive use without proper guidance can negatively impact social skills.

Reference 11 - 0.38% Coverage

The study found that excessive use of smartphones and tablets can negatively impact children's social interactions, leading to reduced face-to-face interactions and potential social competence issues.

Reference 12 - 0.66% Coverage

The conceptualization performances of the three- and five-year-olds supported by an artificial intelligence tool over the tablet computer version also increased

The study does not specifically address the influence of touchscreens on social development, but it suggests that digital tools can be integrated into developmental screening processes.

Reference 13 - 0.29% Coverage

The use of touchscreens, particularly tablets and smartphones, promotes peer interaction and collaboration as children work together to complete tasks.

Reference 14 - 0.40% Coverage

Smartboards, on the other hand, are used in teacher-led activities that resemble classic classroom settings, which may limit peer-to-peer interaction but still support social learning through guided participation.

Reference 15 - 0.32% Coverage

However, the success of this collaboration depends on the children's collaborative orientation and their ability to coordinate their actions and maintain joint attention.

Reference 16 - 0.29% Coverage

The study found that children engaged in physically active, joyful, and cooperative interactions, which helped build social skills and collective agency.

Reference 17 - 0.16% Coverage

The youngest children (3–4 years old) are more interested in manipulating objects.

Reference 18 - 0.63% Coverage

These results are not surprising because children 3 years old show improved control of crayons or markers, enjoy building with blocks, and like manipulate objects, and from 4 years, children begin to cooperate with others, they participate in group activities, they show pride in accomplishments, and they seek frequent adult approval.

Reference 19 - 0.36% Coverage

This shift in interaction may lead to less educationally enriching experiences for toddlers, as their verbalizations and collaborative engagement are reduced compared to reading print books.

Reference 20 - 0.41% Coverage

Specifically, two forms of social interaction—where other children initiated involvement in play (SI1) and where the child playing involved others (SI2)—were significantly correlated with increased engagement levels.

Reference 21 - 0.26% Coverage

The research found that children demonstrated high levels of engagement when they interacted with peers while using digital technology.

Reference 22 - 0.24% Coverage

This engagement was enhanced when children initiated or were involved in peer interactions, which promoted social collaboration.

Reference 23 - 0.26% Coverage

Moreover, the findings highlight that children's self-reflection plays a crucial role in how they engage with digital play and their peers.

Reference 24 - 0.36% Coverage

The use of technology, such as touchscreens, can actively engage children in designated activities, potentially improving their outcomes in terms of engagement and reducing off-task behaviors.

Reference 25 - 0.35% Coverage

However, the paper also highlights the need for further research into appropriate methods of using technology to ensure that it promotes active engagement rather than passive consumption.

Reference 26 - 0.50% Coverage

Therefore, while touchscreens can be beneficial in fostering engagement, their impact on social development, including peer interactions and cooperation, may depend on how they are integrated into learning environments and the nature of the activities they support.

Reference 27 - 0.26% Coverage

The ability to engage more effectively during these activities likely enhances their opportunities for peer interactions and cooperation.

Reference 28 - 0.32% Coverage

However, the study also indicates variability in children's preferences for video versus photos, suggesting that individual choices may impact their engagement levels.

Reference 29 - 0.46% Coverage

The observations indicated that interactions involving the iPad were highly responsive, allowing children to engage in open-ended drawing activities with less personal investment in individual contributions compared to traditional paper drawing.

Reference 30 - 0.37% Coverage

The study highlights that the introduction of iPads in the classroom fosters increased peer collaboration, where pupils proactively share knowledge and assist each other in using the technology.

Reference 31 - 0.33% Coverage

This spontaneous help among peers contributes to improved behavior, especially among boys, as they become more motivated to complete traditional activities to access the iPads.

Reference 32 - 0.27% Coverage

However, it does highlight concerns regarding digital resources potentially encouraging solitary use rather than shared creative interactions.

Reference 33 - 0.34% Coverage

Tasks involving a social demonstration, where an experimenter facilitated actions, significantly improved children's ability to replicate actions compared to ghost demonstrations.

Reference 34 - 0.43% Coverage

The findings indicate that when one member of a dyad (usually the child) was actively engaged with the touchscreen, the overall learning experience was less effective compared to dyads where both members were actively involved.

Reference 35 - 0.44% Coverage

The study highlights the importance of designing interactive displays that accommodate multiple users to foster better cooperation and interaction between children and their parents, ultimately supporting their social development.

Reference 36 - 0.25% Coverage

This interaction encourages discussions and exchanges of ideas, which can lead to more visible and open communication among peers.

Reference 37 - 0.40% Coverage

Additionally, the use of tangible platforms, like the "Quizbot," has been shown to enhance children's ability to reach consensus, divide tasks, and treat each other with respect during collaborative activities.

Reference 38 - 0.46% Coverage

Overall, while the study recognizes the role of digital technology in children's lives, it implies that the quality of social interactions—both with screens and with caregivers—plays a significant role in shaping children's social development.

Reference 39 - 0.42% Coverage

While it highlights that technology plays a significant role in children's lives and can enhance reading enjoyment and outcomes, it does not delve into social development aspects such as peer interactions or cooperation.

Reference 40 - 0.34% Coverage

The study highlights that the use of touchscreens, such as iPads, plays a significant role in young children's social development by facilitating peer interactions and cooperation.

Reference 41 - 0.40% Coverage

Through digital technology, children are able to engage in shared activities, such as imaginary performances and virtual games, which promote social competence as they negotiate and collaborate with one another.

Reference 42 - 0.42% Coverage

This integration of digital technology fosters a collective social situation of development, enabling children to experience a range of emotions, encourage each other, and develop their social skills in a playful context.

Reference 43 - 0.23% Coverage

Overall, touchscreens serve as a valuable tool for enhancing children's social interactions and cooperation with peers.

Reference 44 - 0.50% Coverage

However, the study also highlights that while children were observed using tablets in various ways, the predominant adult gaze was directed towards the screens, which may have limited the range of interactions and play opportunities that foster peer cooperation.

Reference 45 - 0.45% Coverage

By framing the use of tablets primarily as educational tools, the study indicates that certain practices and arrangements of bodies and things were favored, which could reinforce specific social behaviors and hierarchies among children.

Reference 46 - 0.37% Coverage

Ultimately, the research calls for a broader understanding of how these technologies intersect with social inequality and the implications for children's social development in educational settings.

Reference 47 - 0.45% Coverage

While it suggests that social interactions, particularly parent-child interactions, can enhance learning from touchscreen media, it does not specifically discuss the implications for peer interactions and cooperation among young children.

Reference 48 - 0.37% Coverage

The gestures and actions involved in using touchscreens can facilitate shared experiences and discussions among children, potentially enhancing their social interactions and cooperative learning.

Reference 49 - 0.42% Coverage

While it explores media preferences, multitasking behaviors, and parental supervision methods, it primarily focuses on the patterns of media use among children aged three to six and the implications for parental monitoring.

Reference 50 - 0.53% Coverage

The chaotic yet productive nature of collaborative composing with digital puppetry apps showcases how children learn to work together, share materials, and develop strategies for decision-making, ultimately enhancing their social skills through active participation in digital play.

Reference 51 - 0.20% Coverage

The study emphasizes the importance of a rich environment suitable for development during early childhood.

Reference 52 - 0.21% Coverage

It suggests that when parents create a supportive and engaging environment, children's development can thrive.

Reference 53 - 0.56% Coverage

The study implies that excessive use of digital devices, including touchscreens, could detract from opportunities for children to engage in social interactions and cooperative play with peers, as these interactions are crucial for developing social skills during critical developmental periods.

Reference 54 - 0.26% Coverage

phones should not interfere with, and may even boost, social connectedness if phones are used to support people’s nondigital activities.

Reference 55 - 0.29% Coverage

parents feel less attentive and, as a result, less socially connected when they use their smartphones frequently while spending time with their children.

Reference 56 - 0.69% Coverage

Peer interaction was part of the digital literacy activities that involved such mobile technologies as smartphones and tablets, while when using non-mobile technologies, like smartboards, it is shown that the activities were structured more as ‘classic’ classroom activities, primarily guided by the teacher and the didactic material presented through the smartboard.

Reference 57 - 0.45% Coverage

the more frequently used devices (tablets and computers) were most often used for instructional purposes is somewhat promising, suggesting that teachers are aiming to incorporate technology and media use into their curricular objectives,

Reference 58 - 0.20% Coverage

What is more, co-use of the media commonly involved the grandparents’ or great-grandparents’ own devices.

Reference 59 - 0.51% Coverage

the physically active contexts of experimentation with and without timelapse photography and production of Slowmations promoted the children’s more advanced reflections about both the natural phenomenon, i.e. water phases, and creative representations and problem solving.

Reference 60 - 0.43% Coverage

We therefore conclude that the working process, i.e. group discussions followed by experimentation, stimulated recalls and production of Slowmations, is a fruitful setup when working with preschool children and natural phenomena.

Reference 61 - 0.53% Coverage

The analysis of collaborative learning situations supported by ubiquitous computing re- ported on here shows that children's work with constructing and discussing timelapse and stop-motion sequences of science phenomena can be seen to have an impact on the observed communication.

Reference 62 - 0.31% Coverage

This relates to children's understanding of the ethics of turn taking; and demonstrates the trust and social network operating within the children's closed community.

Reference 63 - 0.57% Coverage

Manifestations of social competence can be attributed in part to children's individual differences in temperament and developmental maturity (Howes, 2011), differences that enable some children to be more capable of sharing and engaging in collaborative turn taking than others (Rubin et al., 2006).

Reference 64 - 0.37% Coverage

Not only did children exhibit more collaborative behaviors as sessions shifted away from competition, but in eight of the 20 sessions there was a noticeable collaborative quality from the onset.

Reference 65 - 0.30% Coverage

Children's interactions during those sessions included partners agreeing about decisions, suggesting alternative moves, and assisting with technical concerns.

Reference 66 - 0.51% Coverage

The behaviours of observation and scribing were afforded through the construction of the adult as a skilled user of the resources and the child as an apprentice, who lacked in the skills needed to successfully navigate the digital art-making environment independently.

Reference 67 - 0.22% Coverage

There were some connections made to outside knowledge too as features within the app were related to family experience

Negative

Reference 1 - 0.30% Coverage

The use of touchscreens influences young children's social development by potentially hindering their ability to engage in peer interactions and cooperation.

Reference 2 - 0.19% Coverage

However, excessive screen time might also detract from opportunities for direct peer interactions.

Reference 3 - 0.49% Coverage

However, successful collaboration depends on teacher guidance and structured learning designs

Excessive and unsupervised use of technology can lead to passivity in social interactions and difficulties in peer relationships, potentially resulting in isolation.

Reference 4 - 0.23% Coverage

Higher technology interference was significantly associated with lower response inhibition

and emotional self-regulation.

Reference 5 - 0.76% Coverage

The study highlights that children often prefer digital entertainment over social activities, leading to less face-to-face communication and cooperative experiences

Young children's risk of social–emotional delay was found to increase only when their time spent on game consoles increased, compared with time spent on other popular digital media such as television, computers, tablets, and smartphones.

Reference 6 - 0.53% Coverage

When the study results are examined, generally it is observed that use of mobile technology (television, portable computers, tablets, smart phones and all devices) on weekdays and at weekends did not significantly predict the frequency of children to realize their social skills.

Reference 7 - 0.23% Coverage

However, children who interacted with exhibit screens were less likely to engage in positive interactions immediately after.

Reference 8 - 0.30% Coverage

The article does not specifically address the influence of touchscreen use on young children's social development, including peer interactions and cooperation.

Reference 9 - 0.16% Coverage

However, excessive use without proper guidance can negatively impact social skills.

Reference 10 - 0.35% Coverage

No significant associations were found between proportions of social contexts of use (i.e., solo or co-use) and child internalizing or externalizing behavior in toddlers or preschoolers.

Reference 11 - 0.30% Coverage

This suggests that while touchscreens can support social learning, they may not be as effective as face-to-face interactions in promoting social development.

Reference 12 - 0.66% Coverage

The conceptualization performances of the three- and five-year-olds supported by an artificial intelligence tool over the tablet computer version also increased

The study does not specifically address the influence of touchscreens on social development, but it suggests that digital tools can be integrated into developmental screening processes.

Reference 13 - 0.34% Coverage

This interest is characterized by more interactions and more error processing during the use of the tabletop specifically for the 4- to 6-year-old children than when the stickers.

Reference 14 - 0.26% Coverage

The research found that children demonstrated high levels of engagement when they interacted with peers while using digital technology.

Reference 15 - 0.36% Coverage

The provided context does not specifically address the influence of touchscreens on young children's social development, including their ability to engage in peer interactions and cooperation.

Reference 16 - 0.40% Coverage

The study highlights that higher parent-child technology interference is associated with negative outcomes in social-emotional development, such as increased externalizing and internalizing behavioral problems.

Reference 17 - 0.22% Coverage

These behavioral issues could potentially hinder children's social interactions and cooperative behaviors with peers.

Reference 18 - 0.45% Coverage

The findings suggest that technology interference may disrupt parent-child interactions, which are crucial for developing social skills, but specific effects on peer interactions and cooperation are not explicitly examined in the study.

Reference 19 - 0.32% Coverage

By utilizing technology in an interactive manner, the children demonstrated increased engagement and reduced off-task behaviors, which are critical for social development.

Reference 20 - 0.53% Coverage

The study indicates that there is a need for further research to understand the implications of these changes on peer interactions and cooperation, particularly regarding how touchscreens may facilitate or hinder traditional forms of social engagement and collaborative learning.

Reference 21 - 0.39% Coverage

According to the study, the use of touchscreens, specifically iPads, does not significantly alter young children's social development in terms of their ability to engage in peer interactions and cooperation.

Reference 22 - 0.47% Coverage

The study focuses primarily on the mechanisms of imitation and learning in children using touchscreen tasks, comparing different learning conditions (imitation, emulation, trial-and-error, recall) and examining age-related improvements in these tasks.

Reference 23 - 0.39% Coverage

While the study highlights the role of social learning through imitation and emulation, it does not specifically discuss the broader implications of touchscreen use on social development or peer interactions.

Reference 24 - 0.76% Coverage

While it discusses issues like technoference and its potential effects on child development, it does not specifically examine the influence of touchscreen use on children's social skills or peer interactions

The focus of the research is primarily on children's access to touchscreens, their engagement with stories, and the impact on literacy outcomes, particularly in relation to socioeconomic status.

Reference 25 - 0.41% Coverage

The ability to connect in virtual spaces allows children to maintain friendships and build social relationships even when face-to-face contact is not possible, particularly during situations like the COVID-19 pandemic.

Reference 26 - 0.40% Coverage

The study highlights that parents express concerns regarding the potential negative impact of touchscreen use on young children's social development, particularly in relation to peer interactions and cooperation.

Reference 27 - 0.33% Coverage

Parents worry that excessive time spent on touchscreens may limit opportunities for outdoor play and face-to-face interactions, which are crucial for developing social skills.

Reference 28 - 0.50% Coverage

This suggests that while touchscreen technologies are integrated into children's lives, there is a prevailing apprehension among parents that such technology use might hinder their children's ability to engage socially with peers and cooperate in group settings.

Reference 29 - 0.45% Coverage

While it suggests that social interactions, particularly parent-child interactions, can enhance learning from touchscreen media, it does not specifically discuss the implications for peer interactions and cooperation among young children.

Reference 30 - 0.33% Coverage

For instance, excessive tapping and impulsivity in young children can hinder their understanding of game mechanics, resulting in frustration and reduced learning opportunities.

Reference 31 - 0.35% Coverage

The increased screen time associated with touchscreens may lead to concerns about digital game addiction and reduced face-to-face interactions, which can negatively impact social skills.

Reference 32 - 0.56% Coverage

The study implies that excessive use of digital devices, including touchscreens, could detract from opportunities for children to engage in social interactions and cooperative play with peers, as these interactions are crucial for developing social skills during critical developmental periods.

Reference 33 - 0.34% Coverage

Later, they expressed their disappointment that this game was lost when they got a new computer, thereby showing how much this co-use meant to them both socially and emotionally.

Reference 34 - 0.39% Coverage

There were also instances in which children had trouble navigating tools or program features, and they expressed dismay when a tool didn’t work or when they couldn’t find a feature being used by a tablemate.

Reference 35 - 0.38% Coverage

In such instances, the response varied across students, with some children quitting immediately, others asking for help, and still others trying several strategies to erase undesired marks and blocks.

Reference 36 - 0.51% Coverag

the physically active contexts of experimentation with and without timelapse photography and production of Slowmations promoted the children's more advanced reflections about both the natural phenomenon, i.e. water phases, and creative representations and problem solving.

Reference 37 - 0.23% Coverage

Applications with weak framing and strong classification seemed to increase the children's participation in the activity.

Reference 38 - 0.38% Coverage

As mentioned, one reason for this may be that there were no right and wrong responses in the applications nor were there any time restrictions enabling the pedagogues to be both reactive and proactive.

Reference 39 - 0.13% Coverage

The player stopped other (non-participating) children's interruption.

Reference 40 - 0.34% Coverage

On other occasions, children maneuvered to exclude peer partners from participating by grabbing the device, blocking visibility, and restraining hands so that no moves were possible.

Reference 41 - 0.19% Coverage

The findings show no significant or recurring differences regarding the children's collaborations.

Reference 42 - 0.25% Coverage

Children's willingness or unwillingness to ask their peers for help is almost certainly influenced by the social dynamics among them.

Reference 43 - 0.20% Coverage

Cooperation was not universal but was on roughly equal display in both the paper drawing and iPad scenarios.

Sentiment analysis: The impact of the use of touchscreen on communication

Reference 1 - 1.83% Coverage

Parents in the study reported that children often struggle with verbal expression and instead point or lead adults to what they want, reflecting a decline in language use

We also found that the relationship between young children's increased time on game consoles and social–emotional development was reflected in five subdimensions: self-regulation, compliance, affect, social communications, and interactions with people

This article demonstrates in what ways the use of the smartphone that has replaced the inertia of the conventional desktop has also transformed the shape and content of intergenerational communication between the migrant child, the left-behind grandparents and the migrant mother as the facilitator of digitalized interaction and mediator between the older and the younger generation.

Reference 2 - 0.41% Coverage

Higher parent-child technology interference is associated with fewer verbal and non-verbal interactions, which can hinder the development of communication skills in young children.

Reference 3 - 0.35% Coverage

However, when parents became less engaged, as observed during the activities, children's communication and reasoning abilities may be negatively impacted.

Reference 4 - 0.68% Coverage

However, the study also found that while there is a relationship between looking at stories on a touch screen and communication outcomes, there were no significant associations between other activities children engage in at home, whether using a touch screen or not, and their attainment at age five.

Reference 5 - 0.22% Coverage

Findings revealed caregivers’ mixed feelings about navigating a confusing technology environment.

Reference 6 - 0.28% Coverage

They shared perceived benefits of one-to-one devices, but also shared fears and concerns about young children's device use.

Reference 7 - 0.87% Coverage

The most meaningful engagement through dialogue occurred when the children tried to 'match up' their play and while still on different devices, they were travelling together through the same stations, discussing what they were doing and engaging in imaginative play

At the same time, the iPad activities seemingly help children to avoid criticism, con- flict, sadness, and withdrawal.
